# Synaptic dysfunction of Aldh1a1 neurons in the ventral tegmental area causes impulsive behaviors

**DOI:** 10.1186/s13024-021-00494-9

**Published:** 2021-10-26

**Authors:** Xinyan Li, Wenting Chen, Xian Huang, Wei Jing, Tongmei Zhang, Quntao Yu, Hongyan Yu, Hao Li, Qing Tian, Yumei Ding, Youming Lu

**Affiliations:** 1 Department of Physiology, School of Basic Medicine and Tongji Medical College, Huazhong University of Science and Technology, Wuhan, 4030030 China; 2 Wuhan Center of Brain Science, Huazhong University of Science and Technology, Wuhan, 430030 China; 3 Department of Neurobiology, School of Basic Medicine and Tongji Medical College, Huazhong University of Science and Technology, Wuhan, 430030 China; 4 Department of Pathophysiology, School of Basic Medicine and Tongji Medical College, Huazhong University of Science and Technology, Wuhan, 430030 China; 5 Department of Stomatology, School of Stomatology, Union Hospital, Tongji Medical College, Huazhong University of Science and Technology, Wuhan, 430030 China

**Keywords:** Aldh1a1 neurons, Synaptic circuits, Delay of gratification, Impulsive behaviors, Alzheimer’s disease

## Abstract

**Background:**

Aldh1a1 neurons are a subtype of gamma-aminobutyric acid (GABA) inhibitory neurons that use Aldh1a1 rather than glutamate decarboxylase (GAD) as an enzyme for synthesizing GABA transmitters. However, the behaviors and circuits of this newly identified subtype of inhibitory interneurons remain unknown.

**Methods:**

We generated a mutant mouse line in which cyclization recombination enzyme (CRE) was expressed under the control of the Aldh1a1 promotor (Aldh1a1-CRE mice). Using this mutant strain of mice together with the heterozygous male Alzheimer’s disease (AD) related model mice (APPswe/PSEN1dE9, or AD mice) and a genetically modified retrograde and anterograde synaptic tracing strategy, we have studied a specific synaptic circuit of Aldh1a1 neurons with system-level function and disease progression in AD mice.

**Results:**

We demonstrate that Aldh1a1 neurons encode delay of gratification that measures self-control skills in decision making by projecting inhibitory synapses directly onto excitatory glutamate neurons in the intermediate lateral septum (EGNIS) and receiving synaptic inputs from layer 5b pyramidal neurons in the medial prefrontal cortex (L5PN). L5PN → Aldh1a1 synaptic transmission undergoes long-term potentiation (LTP). Pathway specific inhibition by either genetic silencing presynaptic terminals or antagonizing postsynaptic receptors impairs delay of gratification, resulting in the impulsive behaviors. Further studies show that reconstitution of Aldh1a1-deficient neurons with the expression of exogenous Aldh1a1 (eAldh1a1) restores Aldh1a1 → EGNIS synaptic transmission and rescues the impulsive behaviors in AD mice.

**Conclusions:**

These results not only identify a specific function and circuit of Aldh1a1 neurons but also provide a cellular point of entry to an important but understudied synaptic mechanism for the induction of impulsive behaviors at an early stage of AD.

**Supplementary Information:**

The online version contains supplementary material available at 10.1186/s13024-021-00494-9.

## Background

Inhibitory γ-amino-butyric acid (GABA)-expressing neurons or GABAergic neurons that release GABA as a neurotransmitter make up more than two-thirds of inhibitory neurons [1–3] and are indispensable for the control of network activities in the mammalian brain, including humans [4–8]. GABA in the brain is synthesized by glutamate decarboxylase (GAD), including GAD65 and GAD67, which have traditionally been used as molecular markers to study the structural and functional properties of GABAergic inhibitory neurons [9, 10]. Recent studies have demonstrated that a fraction of GABAergic neurons in the midbrain, particularly the ventral tegmental area (VTA), use aldehyde dehydrogenase 1a1 (Aldh1a1) rather than GAD as an enzyme to synthesize GABA [11–13]. However, the fundamental features such as axon projection patterns, physiological properties, and functions of this newly identified group of neurons in the adult brain are still unknown.

Aldh1a1 is an evolutionarily conserved enzyme for GABA biosynthesis in plants [14, 15] and is co-expressed with tyrosine hydroxylase (TH), which synthesizes dopamine in the brain of rodents [11, 12, 16]. Thus, Aldh1a1 neurons have been previously defined as a subtype of dopaminergic neurons, which are a center for the control of reward-related behaviors and associated diseases of motivation, decision making, and impulsive behaviors [11, 13, 17]. However, how Aldh1a1 neurons integrate information at the VTA and convey it to their synaptic targets for encoding reward states in physiological and pathological conditions is yet to be studied.

In this study, we generated two mutant lines of mice: Aldh1a1-CRE mice, in which CRE was expressed under the control of the Aldh1a1 promoter, and Aldh1a1^−/−^ mice, in which Aldh1a1 in Aldh1a1 neurons was deleted. Using these mutant mice, we were able to perform an integrative study linking the transcriptional profiles and structural properties of Aldh1a1 neurons with their connectivity and system-level functions. We have reported three main findings: 1) Aldh1a1 neurons form a functional circuit by projecting inhibitory synapses directly onto excitatory glutamate neurons in the intermediate lateral septum (EGNIS) and receiving excitatory synaptic inputs directly from layer 5b pyramidal neurons in the medial prefrontal cortex (L5PN); 2) Aldh1a1 neurons encode delay of gratification, as a measure of self-control skills in value-directed decision making depends on a LTP of L5PN → Aldh1a1 synaptic transmission; and 3) dysfunction of Aldh1a1 → ΕGNIS synaptic transmission impairs delay of gratification, resulting in impulsive behaviors in AD mice. This study has, for the first time, provided a specific synaptic and circuitry mechanism for our understanding of how delayed gratification is encoded and identified a promising target for therapeutic intervention of impulsive diseases.

## Materials and methods

### Animals

Male mice at 120 ± 2 days of age were used to avoid potential differences in Aldh1a1 neurons between sexes. Mice were bred and reared under the same conditions in accordance with our institutional guidelines and the Animal Care and Use Committee of the Animal Core Facility at Huazhong University of Science and Technology, Wuhan, China, and housed in groups of three to five mice/cage under a 12 h light-dark cycle, with lights on at 8:00 am, at a constant ambient temperature (21 ± 1 °C) and humidity (50 ± 5%). All behavioral tests were conducted during the light phase of the cycle. For touchscreen-based choice behavioral tests, the mice were maintained on a restricted diet and kept at 90% of their free-feeding body weight during behavioral testing. The animals were randomly allocated to different experimental conditions in this study. To target specifically to Aldh1a1 neurons, we generated Aldh1a1-CRE mice, in which CRE was expressed under the Aldh1a1 promoter (Supplementary Fig. 10a, b).

For the generation of Aldh1a1^−/−^-CRE mice, a P2A-CRE site was inserted downstream of exon 10. The deletion of exons 11–13 eliminated 100 amino acids (401–500) of the C-terminal, which is essential for enzyme function and stability of Aldh1a1 [18]. The vector design for the generation of Aldh1a1-CRE and Aldh1a1^−/−^-CRE is described in detail in Supplementary Fig. 10a, c. The absence of protein products was established by western blot analysis.

Amyloid model mice (APPswe/PSEN1dE9 mice, or AD mice) with a C57BL/6 genetic background were purchased from the Jackson Laboratory (Stock No.: 005864) and housed in the University animal center. In this study, male AD mice at 5 months old of age were used and identified as heterozygous by genotyping with the following primers:

5′- ATGGTAGAGTAAGCGAGAACACG-3’forward for mutant;

5′- GTGTGATCCATTCCATCAGC − 3’forward for wild type;

5′- GGATCTCTGAGGGGTCCAGT − 3′ reverse for common.

### Cell labeling and monosynaptic tracing

To determine the synaptic targets of Aldh1a1 neurons, a high titer (0.1 μl, 8, × 10^12^ genomic particles/ml) of the CRE-recombination-dependent rAAV1/2-TH-DIO-TKGFP virus particles (helper virus) was stereotaxically injected into the VTA of Aldh1a1-CRE mice to express thymidine kinase (TK) in Aldh1a1 neurons. The coordinates of the stereotaxic virus injections were as follows: AP: −3.6, ML: ±0.7, DV: 4.0. The rAAV1/2-TH-DIO-TKGFP virus was generated by insertion of a double *loxP*-flanked inverted TK-2A-GFP sequence immediately downstream of the TH promoter in the rAAV vector, which were co-transfected with AAV helper1 and helper2 mixers (rAAV1/2) into HEK293 cells to generate a high titer of rAAV1/2-TH-DIO-TKGFP virus particles (3 × 10^12^ genomic particles/ml), as described previously [19]. The TH promoter was used because we wanted to express TKGFP specifically in Aldh1a1-expressing dopaminergic neurons. Twelve days after the injection of rAAV1/2-TH-DIO-TKGFP virus particles, 0.05 μl of a high titer (5 × 10^8^ genomic particles/ml) of a genetically modified version of Herpes simplex virus type 1 strain 129 (H129ΔTK-tdT virus), in which TK was deleted, was then injected. The generation of H129ΔTK-tdT virus particles has been described previously [20, 21]. Seven days after the injection of H129ΔTK-tdT virus particles, the mice were sacrificed and fixed. Furthermore, 24 h after fixation, brain sections were imaged under a laser confocal microscope (Zeiss LSM 800, Zeiss). With the assistance of the helper virus, H129ΔTK-tdT transmits anterogradely through Aldh1a1 neurons to their postsynaptic neurons, as described previously [20–22].

To determine the presynaptic neurons of Aldh1a1 neurons, we expressed TVA/G proteins in Aldh1a1 neurons by injecting the rAAV1/2-TH-DIO-TVA/G-GFP virus into the VTA of Aldh1a1-CRE mice. A high titer (0.1 μl of 7 × 10^10^ genomic particles/ml) of the ΔG-rabies virus that encoded tdT (ΔRV) was applied to the same brain region. This injection caused specific labeling of Aldh1a1 neurons and their presynaptic L5PN. Construction and generation of rAAV1/2-TH-DIO-TVA/G-GFP and ΔRV virus particles have been described previously [19, 20, 22].

### Whole-cell patch-clamp recordings with chemogenetics and optogenetics

To investigate synaptic transmission between Aldh1a1 neurons and EGNIS, we injected the rAAV1/2-TH-DIO-Gi-ChR2tdT, rAAV1/2-TH-DIO-TK, and H129ΔTK-FLP virus particles (BrainVTA Co., Ltd., China) into the VTA and the FLP-recombination-dependent rAAV1/2-CaMKIIα-fDIO-GFP virus into the intermediate lateral septum of Aldh1a1-CRE mice, resulting in the expression of Gi-ChR2tdT in Aldh1a1 neurons and GFP under the control of the CaMKIIα promoter in EGNIS. The coordinates of the stereotaxic virus injections were AP: 0.3, ML: ±0.5, DV: 3.0, in the intermediate lateral septum.

The slices were then prepared and transferred to a holding chamber that contained artificial cerebrospinal fluid (ACSF in mM: 124 NaCl, 3 KCl, 26 NaHCO_3_, 1.2 MgCl_2_, 1.25 NaH_2_PO_4_2H_2_O, 10 C_6_H_12_O_6_, and 2 CaCl_2_ at pH 7.4, 305 mOsm) at 32 °C for 30 min. The temperature was maintained at 22 °C for 60 min. A slice was transferred to a recording chamber, which was continuously perfused with oxygenated ACSF (2 ml/min) at 22 °C. We performed whole-cell current-clamp recordings from GFP-expressing EGNIS in the slices, which were visualized under a fluorescent infrared-phase-contrast (IR-DIC) Axioskop 2FS upright microscope equipped with a Hamamatsu C2400-07E infrared camera, as described previously [19, 20, 22, 23]. Inhibitory postsynaptic currents (IPSCs) were evoked by the delivery of blue laser light onto axon fibers of ChR2-expressing Aldh1a1 neurons and inhibited by infusing 5 μM clozapine-N-oxide (CNO) onto the IS through a cannula. The membrane potentials of EGNIS were held at −70 mV. A high Cl^−^ internal recording solution contained (in mM) 150 CsCl, 10 HEPES, 0.2 EGTA, 2 Mg-ATP, 0.3 guanosine triphosphate, and 0.1% biocytin, pH 7.4 (296 mOsm). The external ACSF solution contained 20 μM CNQX (TOCRIS, 0190) throughout the recordings. IPSCs were sensitive to 20 μM bicuculline (TOCRIS, 0130) GABA_A_ receptor antagonist, showing a GABA_A_ receptor-dependent synaptic response.

To record synaptic transmission between L5PN and Aldh1a1 neurons, we expressed Gi-ChR2tdT in L5PN and GFP in Aldh1a1 neurons. Specifically, we first injected rAAV1/2-TH-DIO-TVA/G and ΔRV-FLP virus particles (Taiting Biotechnology Co., Ltd., China) into the VTA of Aldh1a1-CRE mice to express FLP in L5PN. FLP-recombination-dependent rAAV1/2-fDIO-Gi-ChR2tdT virus was injected into layer five of the ventral medial prefrontal cortex (mPFC). This injection caused the expression of Gi-ChR2tdT in Aldh1a1 presynaptic L5PN. The coordinates of the stereotaxic virus injections were AP: 1.9, ML: ±0.5, DV: 3.0. Construction and generation of the rAAV virus particles have been described previously [19, 20, 22].

Next, we performed whole-cell current-clamp recordings of GFP-expressing Aldh1a1 neurons in the slices. Excitatory postsynaptic currents (EPSCs) were evoked by the delivery of blue laser light onto axon fibers of Gi-ChR2tdT-expressing L5PN at a holding potential of −70 mV and inhibited by infusion of 5 μM CNO into ACSF. The internal recording solutions consisted of (in mM) 140 potassium gluconate, 0.05 EGTA, 10 HEPES, 2 Mg-ATP, 0.2 GTP at pH 7.4 with 292 mOsm. The external ACSF solution contained GABA_A_ receptor antagonists, including 20 μM bicuculline (TOCRIS, 0130). EPSCs were sensitive to 20 μM CNQX (TOCRIS, 0190), showing an AMPA receptor-dependent synaptic response. To record NMDA receptor-mediated EPSCs, which were sensitive to 100 μM DL-AP5 sodium salt (TOCRIS, 0105), the holding potential was switched from −70 mV to +60 mV.

### Electrophysiology and optogenetics in vivo

We anesthetized mice with 6% chloral hydrate (0.06 ml/10 g; intraperitoneally) and planted the coated four tetrodes of twisted 17 μm HM-L with platinum-iridium (10% or 20% platinum, #: 100–167, California Fine Wire Company) with the coordinates of AP: −3.6, ML: ±0.7, DV: 3.5-4.0 in VTA, AP: 0.3, ML: ±0.5, DV: 2.7-3.2 in IS, and AP: 1.9, ML: ±0.5, DV: 2.8-3.3 in the mPFC, as described before [19, 20, 22]. We placed the tetrodes directly above the recording site and secured the driver to the skull using jeweler’s screws and dental cement. A jeweler screw was used as the ground electrode. We screened the cells and behaviors daily for each experimental procedure. During the screening procedures, we lowered the tetrodes slowly over several days in steps of 30 μm. For light stimulation of the ChR2-expressing neurons, we planted a bound 20 μm in diameter, unjacketed optical fiber (Inper Co., Ltd., China) in a tetrode-containing silicone tube (166 μm) into the VTA or layer 5b of the mPFC or the intermediate lateral septum. We validated the position of the optic fibers by electrolytic lesions after light stimulation. We applied 473 nm lasers (DPSS laser, Inper Co., Ltd., China) for light activation of targeting neurons or axon fibers. The laser power ranged from 1 to 5 mW/mm^2^ unless otherwise indicated.

Extracellular single units were recorded from Aldh1a1 and L5PNs. The mice were connected to the recording equipment via AC-coupled unity-gain operational amplifiers (Plexon, Dallas, TX, USA). The signals were amplified 4000- to 8000-fold, as described previously [19, 20, 22]. The spikes were recorded at the same time and isolated using a 250 Hz low-pass filter and a 250 Hz high-pass filter of the commercial software OmniPlex (Plexon). Spike sorting was performed offline using graphical cluster-sorting software (Offline Sorter, Plexon). To estimate the quality of the cluster separation, we calculated the isolation distance and L-ratio using Plexon SDK (www.plexon.com/software-downloads/SDK).

To isolate and analyze spike units from individual neuronal types, we calculated the valley-to-peak time and the half-width of the spikes. Spikes in Aldh1a1 neurons and L5PN were identified and distinguished from the cell types in the same brain regions based on the duration of the negative spike, the firing pattern (complex spikes), and the low average firing rate and validated via light activation of ChR2-expressing Aldh1a1 neurons and L5PN. The average firing rate was expressed as the total number of spikes divided by the total length of the recording period.

### Microdialysis in vivo

We anesthetized mice with 6% chloral hydrate (0.06 ml/10 g) and implanted dialysis guide cannula for insertion of the CMA7 dialysis probe in the IS with the following coordinates of AP: 0.3, ML: ±0.5, DV: 3.0 and secured the cannula to the skull using jeweler’s screws and dental cement. Dialysis was performed 24 h after the probe implantation. The perfusion fluid was pumped through the dialysis probe at a rate of 2 μl/min. Samples were collected on ice containing 3.3 μl of dialysate buffer (0.1 M glacial acetic acid, 0.1 mM EDTA; HPLC grade reagent; and 0.12% oxidized l-glutathione, pH at 3.70). Then, 15 μl of the sample were placed in a polypropylene cryogenic vial with 5 μl of 50 nM DA-D4 in 1 mM HCl, 5 μl of 1 M NaHCO_3_, and 25 μl of freshly prepared 1% dansyl chloride solution in acetone. Samples were incubated at 65 °C for 10 min, chilled on ice for 2 min, and then stored in liquid nitrogen until quantification.

Blue laser light was delivered to ChR2-expressing Aldh1a1 neurons when a stable basal value was obtained. Glutamate, GABA, and dopamine were measured using high performance liquid chromatography with fluorescence detection (HPLC-FD, 150 × 4.6 mm, C18, 5 μm particle size column, Agilent Technologies, USA) coupled to a fluorescence detector (excitation wavelength: 340 nm, emission wavelength: 450 nm, RF-10AxL, Shimadzu Japan). The flow rate was 600 μl/min, the pressure was 463 bar, and the column temperature was set to 45 °C.

### Open-field, object recognition

We measured motor activity within clear boxes (100 cm × 100 cm) and outfitted them with photo-beam detectors to monitor horizontal and vertical activity. Data were analyzed using the MED Associates Activity Monitor Data Analysis software. The mice were placed in the corner of the open-field apparatus and allowed to move freely. Behaviors including resting time (s), ambulatory time (s), vertical/rearing time (s), jump time (s), stereotypic time (s), and average velocity (cm/s) were assessed. The mice were not exposed to the chamber prior to the test. The data were recorded for each animal at 30 min intervals, as described previously [20].

To test the performance in the object recognition task, we subjected seven mice per group for two sessions of one trial each: acquisition and retrieval trials. During the acquisition trial, mice were placed in an arena containing two identical objects for 5 min. Mice that did not explore the objects for 20 s within the 5 min period were excluded from further experiments. We defined exploration as a mouse approaching its nose within 1 cm of an object. This approach was associated with looking, sniffing, or touching. The retrieval session was performed 2 h after the acquisition trial. In this trial, we replaced one of the objects presented in the first trial with a novel object. We then placed the mice back in the arena for 5 min and recorded the total time spent exploring each object. New objects were different in shape and color but were made of the same materials and had similar general dimensions. The objects and arenas were thoroughly cleaned with 70% ethanol between the trials. New objects and the positioning of new objects were counterbalanced in all experiments to avoid bias. Motor activity and time spent in active exploration of familiar or novel objects during the retrieval trial were calculated. The recognition index was expressed as the time spent exploring the novel object divided by the total time exploring both objects and multiplied by 100.

### Delay of gratification touchscreen mouse model

We carried out touchscreen behavioral tasks in an automated touchscreen platform, comprising the Bussey-Saksida mouse touchscreen chamber (Lafayette Instrument, US) equipped with a house light, a reward port, holding a reward magazine with an infrared sensor for detection of a mouse entrance into the port, and a touch-sensitive monitor on the front side. All trials in the chamber were mouse initiated and independent of the experimenters. Testing consisted of pre-training, training, and testing sessions, and each behavioral group contained 9 mice.

In the pre-training session, mice were habituated to the apparatus and learned to nose poke to the stimuli presented in one of three windows, and then through several stages to associate the cue touching on the screen with the delivery of a reward (20 μl of chocolate milkshake, Bright Dairy co., Ltd., China) in the reward magazine as described previously [20, 22]. Once a mouse returned to the magazine and retrieved the reward, the magazine light was turned off, and an inter-trial interval of 20 s was initiated. Mice were subjected to the training session after 4 consecutive days (100 min per day, up to 60 trials). If a mouse failed to execute 60 trials within 60 min in the last day, this mouse was excluded from further experiments.

In the training session, mice were subjected to three types of reward learning tasks for 9 consecutive days, with 60 trials per day (one session per day, lasting up to 60 min), as shown in Supplementary Fig. 11. In the first type of learning task, the mice were trained to nose poke a cue symbol (flower) that was randomly displayed for 5 s in one of the three response windows on the touchscreen. Nose-poking this symbol resulted in a small immediate reward (SIR, 5 μl of chocolate milkshake at a 0-3 s delay). In the second type of learning task, mice were trained to nose poke a cue symbol (spider) that was randomly displayed for 5 s in one of the three response windows on the touchscreen. Nose-poking this symbol resulted in a large delayed reward (LDR, 20 μl of chocolate milkshake at a 6-9 s delay). In the third type of learning task, the mice were trained to nose poke a cue symbol (airplane) that was randomly displayed for 5 s in one of the three response windows on the touchscreen. Nose-poking this symbol resulted in a largest long delayed reward (LLR, 30 μl of chocolate milkshake at a 12-15 s delay). Each task consisted of 20 trials per day. After successful training (> 75% accuracy), the mice were subjected to probe trials. All groups of mice equally learned the behavioral performance throughout the training session.

In the probe trials, the mice were subjected to reward choice tasks, in which mice were required to freely choose between three cue symbols (airplane, spider, and flower) that were displayed for 5 s on the touchscreen, as demonstrated in Supplementary Movies 1-6 and Supplementary Fig. 11. Each symbol was associated with a specific reward (SIR, LDR, or LLR). The order of the symbols was randomized from trial to trial. The mice were allowed to poke only one of the three cue symbols in each trial. Each mouse performed 60 trials per day (one session per day, lasting up to 60 min) for 9 consecutive days. All data presented in this study were derived from probe trials.


**Additional file 1.**


**Additional file 2.**


**Additional file 3.**


**Additional file 4.**


**Additional file 5.**


**Additional file 6.**

Definitions: The time from cue presentation on the touchscreen to nose-poking was defined as the reaction time (R.T). Failure to nose-poking within 5 s was defined as an omitted trial. The time from nose-poking to triggering the infrared of the reward port was defined as the reward-collection delay (RCD). The correct collection of a contingency reward (RCD within the reward delay of SIR, LDR, or LLR) after nose-poking was defined as a correct trial. An incorrect collection of the cue reward (either before or after the reward delay of SIR, LDR, or LLR) was defined as an incorrect trial. A warning white noise with 1 s was instantly given to the mouse after an omitted trial or an incorrect trial. The correct score (C.S) was defined as the percentage of the number of correct trials versus the total number of trials. The trials% was defined as the percentage of the number of correct trials versus the total number of trials on each day of the probe trials. Accuracy was defined as the percentage of the number of correct trials versus the total number of trials on each day of SIR, LDR, and LLR separately.

### Delay of gratification T-maze tests

A modified version of an automatic T-maze apparatus that was matte gray in color and consisted of three arms was used. There was one starting arm and two goal arms (Probecare Scientific, Co., Ltd., China) equipped with a starting box at the end of a start arm and a reward (sugar pellets, 14 mg, Bio-Serv) port holding a reward box with an infrared sensor detecting a mouse entrance into the port in each goal box. Two sliding doors were located at the entrance of each goal arm and the reward box for the restriction of a mouse in this goal arm during the delay period after making a choice. The behavioral testing consisted of habituation, training, and testing sessions, and each behavioral group contained 11 mice.

During habituation, the mice were habituated to the T-maze for a total of 5 days. On day one, the sugar pellets were scattered throughout the maze, and on days two and three, the sugar pellets were placed along the two-goal arms, and on days four and five, the sugar pellets were located at the two-goal boxes. The mice were placed in the start box of the maze and allowed to explore the maze for 10 min each day.

In training sessions, mice were allowed to visit one arm only at a given trial: either a large reward arm (LRA with three sugar pellets after a delay of 0-3 s) or a small reward arm (SRA with one sugar pellet after a delay of 0-3 s). After the mouse entered the goal arm, the sliding doors were closed until the delay was completed. Each mouse performed 50 trials (25 LRAs + 25 SIRs) per day (one session per day, lasting up to 60 min) for 5 consecutive days. After successful training, the mice were subjected to testing sessions.

In the testing sessions, the mice were allowed to visit the LRA with three sugar pellets after a delay of 0-3 s or 6-9 s) or SRA (with one sugar pellet only after a delay of 0-3 s). Each mouse performed 50 trials (LRA with three sugar pellets after a delay of 0-3 s in the 1-25 trials and 6-9 s in the 26-50 trials) per day (one session per day, lasting up to 60 min) for 5 consecutive days. To prevent the effects of spatial discrimination, the LRA location was counterbalanced with 50% mice on the left and the other 50% mice on the right. The percentage of LRA visits (LRA %) was defined as the percentage of LRA visiting trials versus the total number of trials on days one, three, and five of the testing sessions.

### Western blots

We expressed GFP in Aldh1a1 neurons and isolated GFP-expressing Aldh1a1 neurons from the VTA of adult mice. In brief, 12 days after the injection of the rAAV1/2-TH-DIO-GFP virus into the VTA of Aldh1a1-CRE mice, the slices were prepared and digested in buffer containing 10 mM Tris-Cl (pH 7.6), 50 mM NaF, 1 mM Na_3_VO_4_, 1 mM edetic acid, 1 mM benzamidine, 1 mM PMSF, 1 mg/10 ml papain, and a mixture of aprotinin, leupeptin, and pepstatin A (10 μg/ml each) for 30 min. Suspended GFP-expressing Aldh1a1 neurons were automatically isolated using an S3e Cell Sorter (Bio-Rad), homogenized, and diluted with a buffer containing 200 mM Tris-Cl (pH 7.6), 8% SDS, and 40% glycerol. The protein concentration was determined using a BCA kit (Pierce, Rockford, IL, USA). The final concentrations of 10% β-mercaptoethanol and 0.05% bromophenol blue were added, and the samples were boiled for 10 min in a water bath. The proteins in the extracts were separated by 10% SDS-PAGE and transferred to nitrocellulose membranes. The blots were scanned using an infrared imaging system (Odyssey, LI-COR). The blots were incubated with the following antibodies: goat anti-C-terminal-Aldh1a1 (1: 2000, Sigma-Aldrich, SAB2500058) and rabbit anti-α-tubulin (1:2000, Abcam, ab18251), and the band densities were quantitatively analyzed using Kodak Digital Science 1D software (Eastman Kodak, New Haven, CT), as described previously [22, 23]. The full-blot images can be found in the additional file (Original blots).

### Immunohistochemistry

The mice were sacrificed by intraperitoneal injection of an overdose of chloral hydrate and were transcardially perfused with 100 mL saline (0.9% w/v NaCl), followed by 4% paraformaldehyde (PFA). The brains were removed and post-fixed in 4% PFA. Sagittal or coronal sections (30 μm) were sliced (Leica Microsystems, Wetzlar, Germany). Immunohistochemistry was performed on free-floating brain sections, as described previously [22–24]. In brief, staining was performed on 30 μm free-floating coronal sections and blocked in 3% normal donkey serum (room temperature for 1 h). For goat antibodies, donkey serum was used. The sections were then incubated in 50 mM Tris-HCl buffer containing 3% donkey serum and 0.3% Triton X-100 with one of the following primary antibodies: rabbit anti-Aldh1a1 (1: 1000, Abcam, ab52492), mouse anti-CaMKIIα (1: 3000, Abcam, ab22609), goat anti-CHAT (1:2000, Millipore, AB144P), mouse anti-GAD67 (1: 1000, Millipore, MAB5406), rabbit anti-TH (1: 1000, Abcam, ab112), and rat anti-CTIP2 (1: 500, Abcam, ab18465) for 24 h. Sections were rinsed with Tris-HCl buffer containing 3% donkey serum and 0.3% Triton X-100 and reacted with Alexa Fluor 488 donkey anti-rabbit, Alexa Fluor 488 donkey anti-mouse, Alexa Fluor 546 donkey anti-rabbit, Alexa Fluor 488 donkey anti-goat, Alexa Fluor 546 donkey anti-mouse, Alexa Fluor 488 donkey anti-rat at room temperature for 1 h. The sections were rinsed, dried, and cover-slipped with a fluorescence mounting medium. The control sections were processed by omitting the primary antisera. Single or double labeling was viewed and imaged with a confocal laser-scanning microscope (Zeiss LSM800 Examiner Z1) and analyzed with a three-dimensional constructor (Image-Pro Plus software). A confocal series of images were taken at 0.5 μm intervals through the region of interest, and optical stacks of 6-12 images were produced for the figures. We quantified the absolute numbers of single, double, or triple labeled cells by sampling every section (image stacks) from the experimental animals, as described previously [19, 20, 22]. For cell counting, the experimenters coded all slides from the experiments before quantitative analysis. Quantification was performed by other experimenters who were unaware of the experimental conditions and treatments, as described previously [19, 20, 23].

### Statistical analysis

All values in the text and figure legends are represented as the mean ± SEM. Unpaired two-tailed Student’s *t*-tests (*t*-test) and one-way analysis of variance (*ANOVA)* and post hoc Bonferroni’s following a two-way ANOVA (BF *ANOV*A) were used when assumptions of normality and equal variance (*F* test) were met (Supplementary Table 1). Statistical significance was accepted at a *p*-value of < 0.05. Power calculations were performed using G*power software version 3.1.9.2 (IDRE Research Technology Group, Los Angeles, USA). The group sizes were estimated based on recent studies and designed to provide at least 80% power with the following parameters: probability of type I error (α) = 0.05, conservative effect size of 0.25, and three to eight treatment groups with multiple measurements obtained per replicate.

## Results

### Genetically mapping Aldh1a1 neurons in adult mice

**Fig. 1:**
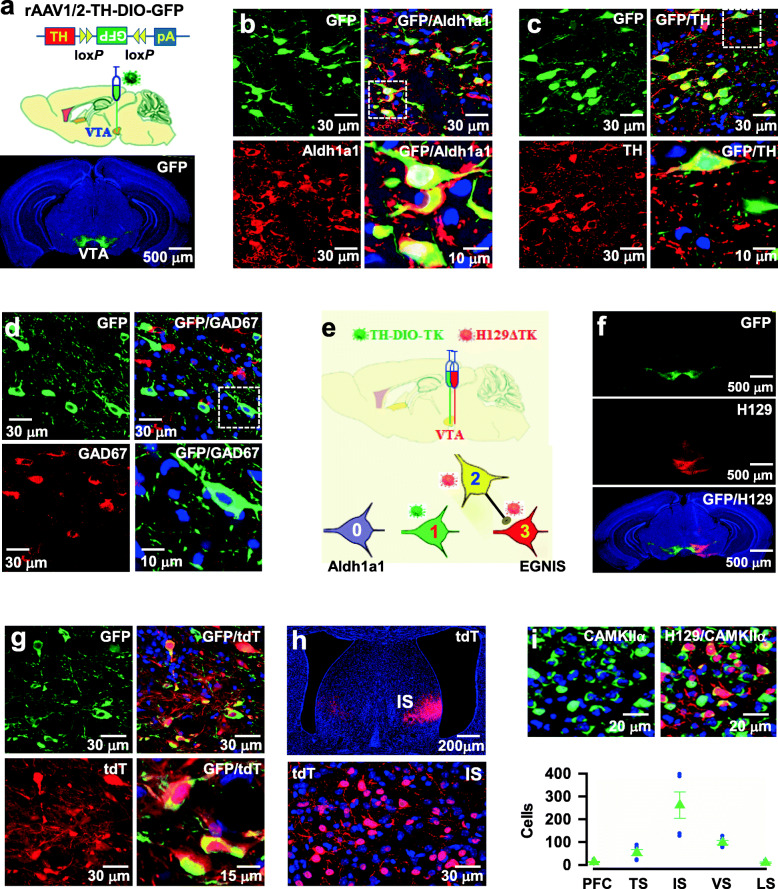
Genetic mapping of Aldh1a1 neurons and their synaptic targets in adult mice. **a,** GFP is expressed in Aldh1a1 neurons (green) after the injection of the rAAV1/2-TH-DIO-GFP virus into the VTA of Aldh1a1-CRE mice. **b,** Representative images show the labeling of GFP-expressing neurons with anti-Aldh1a1 (GFP/Aldh1a1). Higher magnification of a selected area (box) shows the co-labeling of GFP with Aldh1a1. **c,** Representative images show the labeling of GFP-expressing neurons with anti-TH (GFP/TH). Higher magnification of a selected area (box) shows the co-labeling of GFP with TH. **d**, Representative images show the labeling of GFP-expressing neurons with anti-GAD67 (GFP/GAD67). Higher magnification of a selected area (box) shows the absence of GAD67 in GFP-expressing neurons. **e,** Illustration shows the expression of TK (green, 1) in CRE-expressing Aldh1a1 neurons (0) after the injection of rAAV1/2-TH-DIO-TK-GFP virus into the VTA of Aldh1a1-CRE mice. Twelve days later, H129ΔTK-tdT virus was applied. This application caused the expression of tdT (red) in GFP-expressing Aldh1a1 neurons (yellow, 2) and their postsynaptic neurons (red, 3). **f, g,** Low (**f**), and high (**g**) magnifications of the images show the labeling of tdT (red) in GFP-expressing neurons (GFP/ tdT). **h,** Low (top) and high (bottom) magnifications of the images show the expression of tdT (red) in the IS. **i,** The representative images show the labeling of tdT-expressing neurons with anti-CaMKIIα. The plot shows the number of tdT-expressing neurons in the mPFC, the posterior tail of TS, the ventral (VS), and the lateral (LS) striatum from the individual mice (circles) and the averages (mean ± SEM, *n* = 5)

To determine how Aldh1a1 neurons integrate information at the VTA and convey it to their synaptic targets for encoding specific behaviors, we generated a CRE mouse line (Aldh1a1-CRE) that allows selective access to Aldh1a1 neurons in the adult brain. To validate the recombination potential of the Aldh1a1-CRE driver, we applied the rAAV1/2-TH-DIO-GFP reporter virus, in which enhanced GFP was expressed under the control of the TH promoter and CRE recombination, into the VTA of Aldh1a1-CRE mice (Fig. 1a). This application caused the expression of GFP exclusively in Aldh1a1 neurons, with no expression in the other brain regions (971 ± 104 GFP^+^ cells per mouse, mean ± SEM, *n* = 5 mice, Fig. 1a-c and Supplementary Fig. 1a, b). GFP in Aldh1a1 neurons was at a high level in a pattern that was qualitatively similar to Aldh1a1 protein, as ~ 92 ± 8% GFP^+^ cells were co-labeled with antibodies against Aldh1a1, a total of 1072 ± 101 Aldh1a1^+^ cells in the VTA per mouse were counted, of which 891 ± 109 cells were co-labeled with GFP (GFP^+^Aldh1a1^+^, mean ± SEM, *n* = 5 mice, Fig. 1b and Supplementary Fig. 1b), showing the specific labeling of Aldh1a1 neurons.

Most GFP^+^ cells (95 ± 9.2%, mean ± SEM, *n* = 5 mice) were co-labeled with anti-TH, a dopaminergic cell marker. A total of 2454 ± 320 TH^+^ cells in the VTA per mouse were found, of which 919 ± 78 cells were co-expressed with GFP (GFP^+^TH^+^, mean ± SEM, *n* = 5 mice, Fig. 1c and Supplementary Fig. 1a, b), indicating that ~ 37% of the TH^+^ cells were labeled with GFP in the VTA. This finding is consistent with those of previous studies, in which ~ 32% of TH^+^GFP^+^ cells were reported [13], but in an early study, only ~ 25% TH + GFP+ cells were identified [11]. The discrepancy among these studies could be due to the differences in CRE recombination efficiencies, virus infectious titers, and antibody affinities. Notably, GFP^+^ cells lacked the expression of GAD67, which marks a classical GABA inhibitory cell type (Fig. 1d).

### Aldh1a1 neurons form GABA inhibitory synapses with EGNIS

To gain insight into how Aldh1a1 neurons regulate behaviors, we first examined their synaptic targets using an anterograde synaptic tracing technique [21]. A genetically modified version of Herpes simplex anterograde virus encoding membrane-targeted tdT (H129ΔTK-tdT) and the rAAV1/2-TH-DIO-TK/GFP virus was injected into the VTA of Aldh1a1-CRE mice (Fig. 1e-g). This injection caused the expression of tdT in Aldh1a1 neurons (tdT^+^, Fig. 1f, g and Supplementary Fig. 1c, d) and their direct targets, located in the intermediate lateral septum (IS, 262 ± 58 tdT^+^ cells, mean ± SEM, *n* = 5; Fig. 1h) and the striatum, including the posterior tail of the striatum (TS, 54 ± 14 tdT^+^ cells, mean ± SEM, *n* = 5), lateral (LS, 10 ± 3 tdT^+^ cells, mean ± SEM, *n* = 5), and ventral striatum (VS, 98 ± 9 tdT^+^ cells, mean ± SEM, *n* = 5, Fig. 1i), confirming conventional tracing [11, 25]. A pathway from Aldh1a1 neurons to the VS controls alcohol consumption [11]. However, synaptic properties and behavioral functions from Aldh1a1 neurons to the IS are yet to be studied.

Fluorescence labeling with antibodies against Ca^2+^/calmodulin-dependent kinase IIα (CaMKIIα), which marks excitatory glutamate neurons, revealed that the majority of tdT^+^ neurons were labeled with CaMKIIα (88 ± 8.7% of tdT^+^ cells were tdT^+^/CaMKIIα^+^; 262 ± 58 tdT^+^ versus 231 ± 25 tdT^+^/CaMKIIα^+^ cells; mean ± SEM, *n* = 5 mice, Fig. 1i and Supplementary Fig. 1d). Very few tdT^+^ neurons were labeled with ChAT (8 ± 2.4 tdT^+^/ChAT^+^ cells; mean ± SEM, *n* = 5 mice) and GAD67 (5.8 ± 1.7 tdT^+^/GAD67^+^ cells; mean ± SEM, *n* = 5 mice, Supplementary Fig. 1d). Thus, Aldh1a1 neurons directly innervate excitatory glutamate neurons in the IS (EGNIS).

**Fig. 2:**
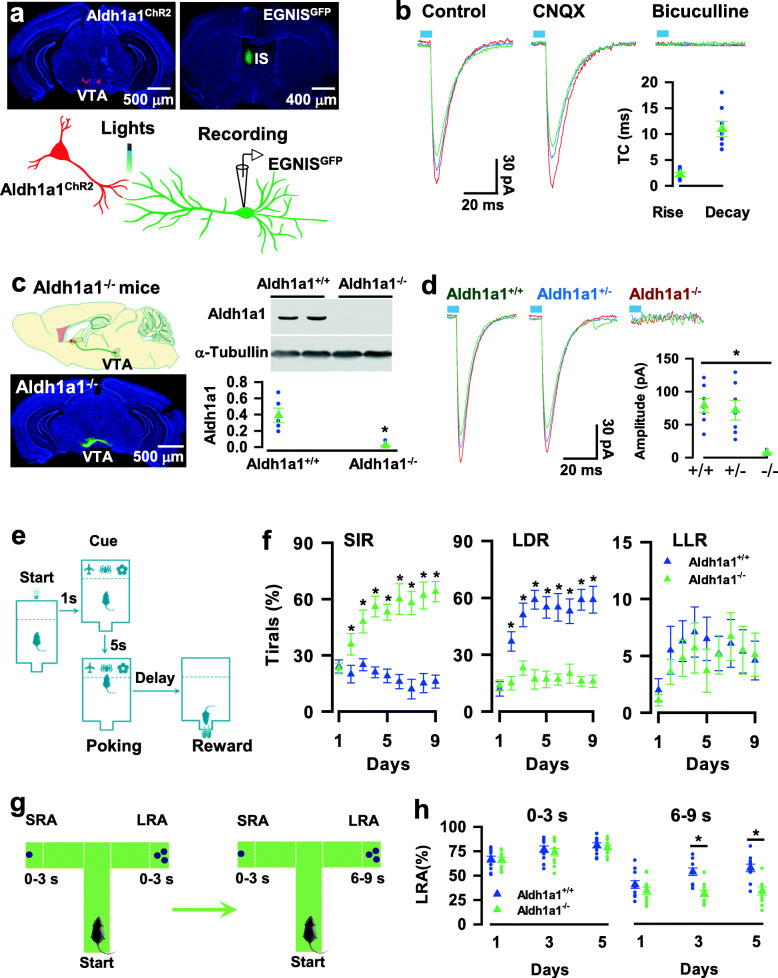
Deletion of Aldh1a1 in Aldh1a1 neurons impairs delay of gratification. **a,** Representative images (top) show the expression of ChR2 (left, red) in Aldh1a1 neurons and GFP in EGNIS (right, green), respectively. The illustration (bottom) shows whole-cell patch-clamp recordings from GFP-expressing EGNIS (R) and laser stimulation of ChR2-expressing Aldh1a1 synaptic terminals (S). **b,** Three representative IPSCs were blocked by bicuculline but not CNQX. The plot shows the rise and decay time constants (T.C) of the individual IPSCs (blue circles) and the averages (green triangles, mean ± SEM, *n* = 7 recordings/5 mice). **c,** The generation of Aldh1a1^−/−^ mice by selective deletion of Aldh1a1 in Aldh1a1 neurons. Representative blots from two pairs of Aldh1a1^+/+^ and Aldh1a1^−/−^ mice. The plot shows the normalized Aldh1a1 protein levels from the individuals (blue circles) and the averages per group (green triangles, mean ± SEM, *n* = 5 assays/5 mice/group). **d,** Aldh1a1 deletion completely blocked synaptic transmission from Aldh1a1 neurons to EGNIS. Three representative traces show the recordings of the evoked IPSCs from Aldh1a1^+/+^, Aldh1a1^+/−^, and Aldh1a1^−/−^ mice. The plot shows the mean amplitudes of the individual IPSCs (blue circles) and the averages per group (green triangles, mean ± SEM, *n* = 7 recordings/3 mice/group). **e**, A touchscreen mouse model for delay of gratification tests, comprised of a series of the reward choice tasks, in which mice are required to choose a SIR (5 μl of chocolate milkshake at a 0-3 s delay) or a LDR (20 μl chocolate milkshake at a 6-9 s delay) versus a LLR (30 μl of chocolate milkshake at a 12-15 s delay). **f**, Deletion of Aldh1a1 decreases the behavioral preference for LDR. Plots show the percentage of the correct trials with the behavioral preference for SIR, LDR, or LLR of Aldh1a1^+/+^ (blue) and Aldh1a1^−/−^ (green) mice at each day of the probe trials (mean ± SEM, *n* = 9 mice/group). **g**, A T-maze mouse model for delay of gratification tests, consisting of the SRA at a delay of 0-3 s and LRA at a delay of 0-3 s or 6-9 s. **h**, Deletion of Aldh1a1 reduces the percentage of LRA visits after a delay of 6-9 s. The plot shows the percentage of LRA visits at a delay of 0-3 s or 6-9 s from individual (circles) Aldh1a1^+/+^ (blue) or Aldh1a1^−/−^ (green) mice and their averages per group (triangles, mean ± SEM, *n* = 11 mice/group) at day one, three and five of the testing sessions. All statistical data are summarized in Supplementary Table 1

To determine whether Aldh1a1 neurons form functional synapses with EGNIS, we applied the rAAV1/2-TH-DIO-TK-IRES-ChR2 virus to the VTA of Aldh1a1-CRE mice, resulting in the expression of TK and channel rhodopsin-2-H134R (ChR2), a modified version of a light-gated ion channel, in Aldh1a1 neurons (Fig. 2a). Twelve days after the injection, the H129ΔTK-FLP virus was injected into the VTA, leading to the expression of the FLP recombination enzyme specifically in EGNIS. FLP recombination-dependent rAAV1/2-fDIO-GFP virus was injected into the IS, causing the expression of GFP specifically in Aldh1a1-targeted EGNIS. We then performed whole-cell voltage-clamp recordings from GFP-expressing EGNIS in brain slices (Fig. 2b). Blue laser light illumination of the axon fibers of ChR2-expressing Aldh1a1 neurons with a brief pulse reliably evoked IPSCs, with a short latency. The evoked IPSCs that were recorded in a high Cl^−^ intracellular solution were completely blocked by 20 μM bicuculline (Fig. 2b), but not α-amino-3-hydroxy-5-methylisoxazole-4-propionic acid (AMPA)-receptor antagonist 6-cyano-7-nitroquinoxaline-2,3-dione (CNQX), showing GABA inhibitory synaptic transmission.

### Aldh1a1 → EGNIS synaptic transmission via Aldh1a1

To investigate Aldh1a1-dependence of GABA inhibitory Aldh1a1 → EGNIS synaptic transmission, we genetically deleted Aldh1a1 in Aldh1a1 neurons of adult mice by generating Aldh1a1^−/−^-CRE mutant mice, resulting in the specific deletion of Aldh1a1 and expression of CRE in Aldh1a1 neurons of adult mice (*n* = 5, Fig. 2c). Notably, the deletion of Aldh1a1 effectively blocked Aldh1a1 → EGNIS synaptic transmission (*n* = 7 recordings/3 mice, Fig. 2d).

To further determine Aldh1a1-dependence of Aldh1a1 → EGNIS synaptic transmission, we analyzed the extracellular concentrations of GABA, dopamine, and glutamate neurotransmitters by using brain microdialysis in Aldh1a1^−/−^-CRE mice, in which Aldh1a1 neurons were expressed with ChR2. In control mice (Aldh1a1-CRE mice), the extracellular concentrations of GABA and dopamine were dramatically elevated from the baseline after the delivery of blue laser light onto ChR2-expressing Aldh1a1 neurons, whereas glutamate was unchanged (Supplementary Fig. 2a, b). Significantly, we found that the deletion of Aldh1a1 inhibited GABA release without altering dopamine (Supplementary Fig. 2c, d). Thus, Aldh1a1 neurons co-release GABA with dopamine. Deletion of Aldh1a1 selectively eliminated GABA inhibitory transmitter release from Aldh1a1 neurons, suggesting that it produced no effect on the distribution and synaptic targets of Aldh1a1 neurons.

### Aldh1a1 deletion induces impulsive behaviors

To explore the roles of Aldh1a1 neurons and their inhibitory synaptic output to EGNIS in behaviors, we examined the phenotypes of Aldh1a1^−/−^ mice using various behavioral tests. Compared with wild-type littermates (Aldh1a1^+/+^ mice), Aldh1a1^−/−^ mice performed normally throughout the light-dark phases (*n* = 7 mice per group, Supplementary Fig. 3a-c), the elevated plus-maze test (*n* = 7 mice per group, Supplementary Fig. 3d), and novel object recognition, but the performance did not differ between groups (*n* = 7 mice per group, Supplementary Fig. 3e).

Aldh1a1 neurons constitute ~ 37% of dopaminergic neurons in the VTA, which are implicated in reward, value, motivational states, and impulsive behaviors [26–30]. Thus, we hypothesized that the deletion of Aldh1a1 might affect rewarding behavior. To test this idea, we designed a touchscreen mouse model for examining delay-based decision making, in which mice were trained to freely choose among rewards with a variety of different sizes and delays (Fig. 2e). These included a SIR (5 μl of chocolate milkshake at a 0-3 s delay) and a LDR (20 μl of chocolate milkshake at a 6-9 s delay) versus a LLR (30 μl of chocolate milkshake at a 12-15 s delay). Overall, mice were able to perceive differently sized and delayed rewards as having different values across all of the probe trials; the C. S (38 ± 5.9 in Aldh1a1^+/+^ mice versus 38.1 ± 3.7 in Aldh1a1^−/−^ mice at day one and 79.6 ± 6.7 in Aldh1a1^+/+^ mice versus 85.1 ± 8.5 in Aldh1a1^−/−^ mice at day nine of the probe trials; mean ± SEM, *n* = 9 mice per group, Supplementary Fig. 4a), and R. T (3.7 ± 0.29 in Aldh1a1^+/+^ mice versus 3.6 ± 0.15 in Aldh1a1^−/−^ mice at day one and 1.8 ± 0.25 in Aldh1a1^+/+^ mice versus 1.9 ± 0.33 in Aldh1a1^−/−^ mice at day nine of the probe trials, mean ± SEM, *n* = 9 mice per group, Supplementary Fig. 4a) were comparable between phenotypes throughout the probe trials, indicating the normality of reward learning and motivation.

Next, we analyzed the behavioral preferences for SIR, LDR, and LLR. Aldh1a1^+/+^ mice initially displayed a preference for SIR over LDR on day one of the probe trials (24 ± 3.5% for SIR versus 12 ± 3.8% for LDR; mean ± SEM, *n* = 9 mice per group, Fig. 2f). With increasing experience, the Aldh1a1^+/+^ mice shifted the behavioral options. After 3 days of the probe trials, Aldh1a1^+/+^ mice exhibited a strong preference for LDR over SIR (51 ± 6.2% for LDR versus 25 ± 3.2% for SIR; mean ± SEM, *n* = 9 mice per group, Fig. 2f). This behavioral preference for LDR is referred to as delay of gratification, which measures self-control skills in value-directed decision making [31–37]. Compared to the Aldh1a1^+/+^mice, the Aldh1a1^−/−^ mice were severely impaired in the tests, with a complete loss of the behavioral preference for LDR on day three of the probe trials (23 ± 3.8% in Aldh1a1^−/−^ mice versus 51 ± 6.2% in Aldh1a1^+/+^ mice for LDR and 48 ± 6.1% in Aldh1a1^−/−^ mice versus 25 ± 3.2% in Aldh1a1^+/+^ mice for SIR, mean ± SEM, *n* = 9 mice per group, Fig. 2f). Overall, the percentage of correct trials in SIR, LDR, and LLR did not differ among groups (Accuracy, mean ± SEM, *n* = 9 mice per group, Supplementary Fig. 4b), indicating that Aldh1a1 deletion impairs delay of gratification, resulting in impulsive behaviors. As noted, although Aldh1a1^+/+^ mice displayed a strong preference for LDR over SIR, this preference was lost in LLR. This finding is consistent with the notion that as a delay to a large reward becomes longer, animals usually discount the value of this large reward, biasing their choice toward a relatively smaller, available reward (LDR), referred as time discounting [38].

To further examine the specific role of Aldh1a1 in delay of gratification, we used a T-maze mouse model of delay-based decision making tests (Fig. 2g), in which mice were allowed to freely visiting a large reward arm (LRA with three sugar pellets after delays from 0-3 to 6-9 s) versus a small reward arm (SRA with one sugar pellet after a delay of 0-3 s). Both Aldh1a1^−/−^ and Aldh1a1^+/+^ mice performed similarly in completion of the training schedule, in which mice were allowed to visit only one arm at a given trial, either a LRA or a SRA at the same delay (0-3 s, Supplementary Fig. 4c), and the two groups displayed no significant interaction in the preference for the LRA when the delays at both the LRA and SRA were 0-3 s during the testing sessions (66.1 ± 3.1% in Aldh1a1^+/+^ mice (blue) versus 66.4 ± 2.7% in Aldh1a1^−/−^ mice (green) at day one; 77 ± 3.4% in Aldh1a1^+/+^ mice versus 74.3 ± 3.8% in Aldh1a1^−/−^ mice at day three; 80.1 ± 2.6% in Aldh1a1^+/+^ mice versus 79.2 ± 2.3% in Aldh1a1^−/−^ mice at day five; mean ± SEM, *n* = 11 mice per group, Fig. 2h). Hence, Aldh1a1 deletion produces no effect on a sensitivity to reward magnitude. However, when the delay time of the LRA was elongated from 0-3 s to 6-9 s, the Aldh1a1^−/−^ mice displayed a significant reduction in the frequency of LRA visits (39 ± 3.8% in Aldh1a1^+/+^ mice versus 33 ± 3.6% in Aldh1a1^−/−^ mice on day one; 52 ± 3.8% in Aldh1a1^+/+^ mice versus 30 ± 3% in Aldh1a1^−/−^ mice at day three; 56 ± 3.7% in Aldh1a1^+/+^ mice versus 32 ± 4.1% in Aldh1a1^−/−^ mice at day five; mean ± SEM, *n* = 11 mice per group, Fig. 2h), confirming that Aldh1a1 neurons play an essential role in delay of gratification and genetic deletion of Aldh1a1 causes impulsive behaviors.

**Fig. 3:**
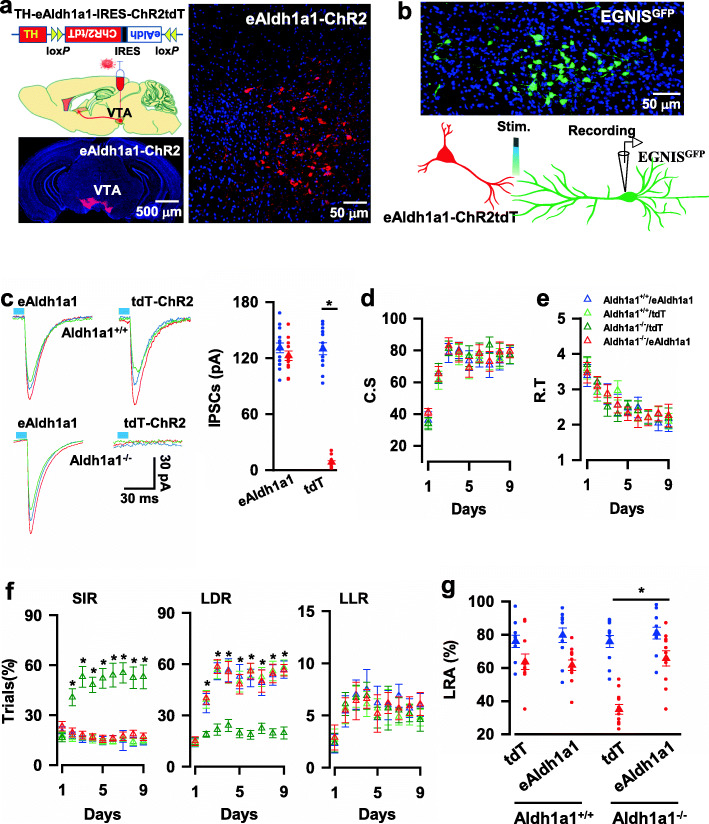
Expression of exogenous Aldh1a1 in Aldh1a1-lacking neurons rescues delay of gratification. **a,** The expression of eAldh1a1-ChR2 in Aldh1a1^−/−^ neurons (Aldh1a1^eAldh1a1-ChR2^) and GFP in EGNIS (EGNIS^GFP^) in Aldh1a1^−/−^ mice. **b**, The illustration shows whole-cell patch-clamp recordings from EGNIS^GFP^. Synaptic currents are evoked by blue laser light stimulation on Aldh1a1^eAldh1a1-ChR2^ axon fibers. **c**, Three representative IPSCs are recorded from EGNIS^GFP^ neurons in the slices from Aldh1a1^+/+^ (blue) or Aldh1a1^−/−^ (red) mice with the expression of eAldh1a1-ChR2 or tdT-ChR2 in Aldh1a1 neurons. The plot shows the mean amplitude of the evoked IPSCs in the presence of 20 μM CNQX at a holding potential of −70 mV from the individual slices (circles) and the averages per group (triangles, mean ± SEM, *n* = 12 recordings/six mice/group). **d**, The plot shows the average C. S of Aldh1a1^+/+^ with the expression of eAldh1a1 (blue) or tdT (green) and Aldh1a1^−/−^ mice with the expression of eAldh1a1 (red) or tdT (dark green) at each day of the probe trials (mean ± SEM, *n* = 9 mice/group). **e**, The plot shows the average R. T of Aldh1a1^+/+^ with the expression of eAldh1a1 (blue) or tdT (green) and Aldh1a1^−/−^ mice with the expression of eAldh1a1 (red) or tdT (dark green) at each day of the probe trials (mean ± SEM, *n* = 9 mice/group). **f**, The plots show the percentage of the correct trials with the behavioral options for SIR, LDR, or LLR of Aldh1a1^+/+^ with the expression of eAldh1a1 (blue) or tdT (green) and Aldh1a1^−/−^ mice with the expression of eAldh1a1 (red) or tdT (dark green) at each day of the probe trials (triangles, mean ± SEM, *n* = 9 mice/group). **g**, The plot shows the percentage of LRA visits at a delay of 0-3 s (blue) or 6-9 s (red) from individual (circles) Aldh1a1^+/+^ with the expression of eAldh1a1 or tdT and Aldh1a1^−/−^ mice with the expression of eAldh1a1 or tdT and their averages per group (triangles, mean ± SEM, *n* = 11 mice/group) at day three of the testing sessions. All statistical data are summarized in Supplementary Table 1

To determine a sufficient role of Aldh1a1 in delay of gratification, we reconstituted Aldh1a1-deficient neurons with the expression of exogenous Aldh1a1 (eAldh1a1), and this reconstitution completely restored GABA inhibitory Aldh1a1 → EGNIS synaptic transmission (131 ± 5.2 pA in Aldh1a1^+/+^ mice expressing eAldh1a1, 130 ± 6.4 pA in Aldh1a1^+/+^ mice expressing tdT, 120 ± 5.1 pA in Aldh1a1^−/−^ mice expressing eAldh1a1 versus 8.6 ± 1.6 pA in Aldh1a1^−/−^ mice expressing tdT, mean ± SEM, *n* = 12 recordings/6 mice, Fig. 3a-c) and rescued delay of gratification. The expression of eAldh1a1 did not affect the values of C. S (78.5 ± 6% in Aldh1a1^+/+^ mice expressing eAldh1a1 (blue), 81.3 ± 4.6% in Aldh1a1^+/+^ mice expressing tdT (green), 83 ± 5.1% in Aldh1a1^−/−^ mice expressing eAldh1a1 (red) versus 81.4 ± 4.6% in Aldh1a1^−/−^ mice expressing tdT (dark green) at day three of the probe trials, mean ± SEM, *n* = 9 mice per group, Fig. 3d), R. T (2.87 ± 0.29 in Aldh1a1^+/+^ mice expressing eAldh1a1, 2.88 ± 0.25 in Aldh1a1^+/+^ mice expressing tdT, 2.87 ± 0.26 in Aldh1a1^−/−^ mice expressing eAldh1a1 versus 2.5 ± 0.26 in Aldh1a1^−/−^ mice expressing tdT at day three of the probe trials, mean ± SEM, *n* = 9 mice per group, Fig. 3e), and Accuracy (Supplementary Fig. 4d). But it caused a strong behavioral preference for LDR (55.9 ± 5.0 trials% in Aldh1a1^+/+^ mice expressing eAldh1a1, 56.8 ± 5.3 trials% in Aldh1a1^+/+^ mice expressing tdT, 58.6 ± 4.1 trials% in Aldh1a1^−/−^ mice expressing eAldh1a1 versus 21.7 ± 3.3 trials% in Aldh1a1^−/−^ mice expressing tdT at day three of the probe trials, mean ± SEM, *n* = 9 mice per group, Fig. 3f) and significantly increased the incidents of LRA visits (61.6 ± 3.2% in Aldh1a1^+/+^ mice expressing eAldh1a1, 63.7 ± 4.7% in Aldh1a1^+/+^ mice expressing tdT, 65.7 ± 4.6% in Aldh1a1^−/−^ mice expressing eAldh1a1 versus 35 ± 3% in Aldh1a1^−/−^ mice expressing tdT at day three of the probe trials, mean ± SEM, *n* = 11 mice per group, Fig. 3g). Together, Aldh1a1 neurons act as necessary and sufficient mediators for encoding delay of gratification.

### Aldh1a1 → EGNIS synaptic dysfunction induces impulsive behaviors

**Fig. 4:**
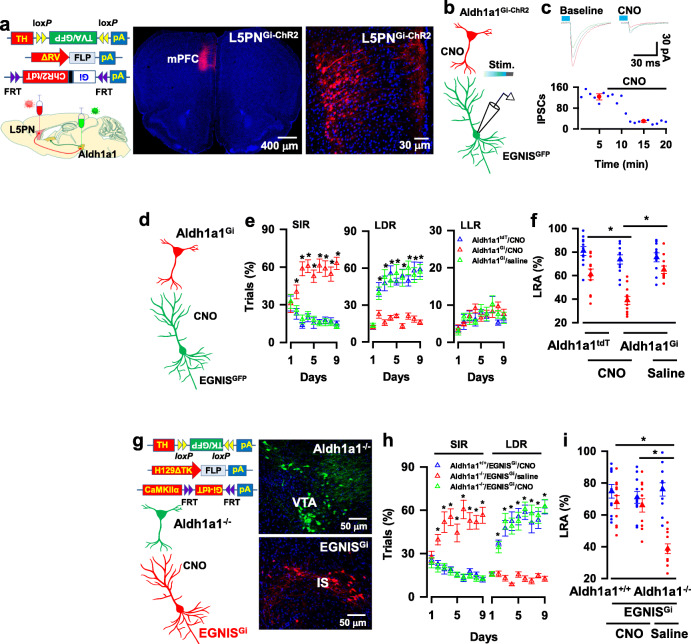
Synaptic transmission^Aldh1a1 → EGNIS^ mediates delay of gratification. **a,** The expression of Gi-ChR2 and GFP in Aldh1a1 neurons (Aldh1a1^Gi-ChR2^) and EGNIS (EGNIS^GFP^), respectively. **b**, The illustration shows IPSCs were recorded from GFP-expressing EGNIS and evoked by the delivery of blue laser light onto axon terminals of Aldh1a1^Gi-ChR2^ neurons. Chemogenetic inhibition of Aldh1a1 axon terminals was achieved by infusing CNO at a concentration of 5 μM. **c**, Chemogenetic inhibition of synaptic transmission^Aldh1a1 → EGNIS^. The plot shows the mean amplitude of the evoked IPSCs versus the time of the individual recordings (blue) and the averages (red, mean ± SEM, *n* = 12 recordings/6 mice) of the evoked IPSCs without (baseline) or with CNO. Three representative traces are the averages of 5 min recordings from the baseline or the presence of CNO. **d**, Aldh1a1^tdT^ or Aldh1a1^Gi^ mice, in which tdT or Gi was expressed in Aldh1a1 neurons were infused of 1 μl of CNO at a concentration of 500 μM or saline into IS 30 mice before behavioral testing, resulting in synaptic terminal silencing. **e**, Chemogenetic inhibition of Aldh1a1 synaptic outputs reduces the behavioral preference for LDR. The plots show the percentage of the correct trials with the behavioral options for SIR, LDR, or LLR of Aldh1a1^tdT^ mice with CNO (blue), Aldh1a1^Gi^ mice with CNO (red), or Aldh1a1^Gi^ mice with saline (green) at each day of the probe trials (mean ± SEM, *n* = 9 mice per group). **f,** The chemogenetic inhibition of Aldh1a1 synaptic projections reduce the percentage of LRA visits. The plot shows the percentage of LRA visits at a delay of 0-3 s (blue) or 6-9 s (red) from the individual (circles) Aldh1a1^tdT^ mice with CNO or Aldh1a1^Gi-^ mice with CNO or Aldh1a1^Gi^ mice with saline and their averages per group (triangles, mean ± SEM, *n* = 11 mice/group) at day three of the testing sessions. **g**, EGNIS was expressed with Gi (EGNIS^Gi^) in Aldh1a1^−/−^ mice. **h**, Chemogenetic inhibition of postsynaptic EGNIS in Aldh1a1^−/−^ mice increases the behavioral preference for LDR. The plot shows the percentage of the correct trials with the behavioral options for SIR or LDR of Aldh1a1^+/+^ mice or Aldh1a1^−/−^ mice with the expression of Gi in EGNIS (EGNIS^Gi^) infused with CNO (blue or green) or saline (red) at each day of the probe trials (mean ± SEM, *n* = 9 mice per group). **i,** Chemogenetic inhibition of postsynaptic EGNIS in Aldh1a1^−/−^ mice increases the percentage of LRA visiting. The plot shows the percentage of LRA visits at a delay of 0-3 s (blue) or 6-9 s (red) from individual (circles) Aldh1a1^+/+^ mice or Aldh1a1^−/−^ mice with the expression of Gi in EGNIS (EGNIS^Gi^) infused with CNO or saline and their averages per group (triangles, mean ± SEM, *n* = 11 mice/group) at day three of the testing sessions. All statistical data are summarized in Supplementary Table 1

Next, we determined whether delay of gratification was mediated by Aldh1a1 → EGNIS synaptic transmission. This was investigated by genetically silencing the presynaptic terminals of Aldh1a1 neurons. We genetically engineered Aldh1a1 neurons by expressing an inhibitory G-protein coupled receptor, hM4Di, with ChR2 (Gi-ChR2, Aldh1a1^Gi-ChR2^ neurons, Fig. 4a). The Gi agonist CNO at a concentration of 5 μM was applied to the slices from mice, in which Gi-ChR2 and GFP were expressed in Aldh1a1 neurons and EGNIS, respectively (Fig. 4b). Application of CNO decreased excitability of Aldh1a1 neurons (Supplementary Fig. 5a) and caused a marked reduction of the evoked IPSC to 23 ± 1.5% of baseline (123 ± 12 pA at baseline versus 29 ± 3.7 pA in CNO, mean ± SEM, *n* = 11 recordings/6 mice, Fig. 4b, c), confirming the efficacy of synaptic terminal inhibition.

We determined the behavioral effect of terminal inhibition by infusing 1 μl of CNO into the IS of Aldh1a1^Gi+^ mice 30 min before the behavioral tests (Fig. 4d). As shown in Fig. 4e, it significantly decreased the behavioral preference for LDR (47.6 ± 6 trials% in Aldh1a1^tdT^ mice with CNO mice (blue), 15.7 ± 2.3 trials% in Aldh1a1^Gi^ mice with CNO (red) versus 52.6 ± 6.2 trials% in Aldh1a1^Gi^ mice with saline (green) at day three of the probe trials; mean ± SEM, *n* = 9 mice per group) and increased the preference for SIR (13.6 ± 2.2 trials% in Aldh1a1^tdT^ mice with CNO mice, 58.6 ± 5.5 trials% in Aldh1a1^Gi^ mice with CNO versus 18.3 ± 3.5 trials% in Aldh1a1^Gi^ mice with saline at day three; mean ± SEM, *n* = 9 mice per group). Synaptic terminal silencing of Aldh1a1 neurons did not affect the values of C. S, R. T, and Accuracy (Supplementary Fig. 5b, c), but it significantly decreased the incidence of visits for LRA (61 ± 4.6% in Aldh1a1^tdT^ mice with CNO mice, 39 ± 3.4% in Aldh1a1^Gi^ mice with CNO versus 65 ± 3.1% in Aldh1a1^Gi^ mice with saline; mean ± SEM, *n* = 11 mice per group, Fig. 4f). Together, these findings indicate that Aldh1a1 neurons control delay of gratification, at least in part, through synapsing with EGNIS.

Next, we determined whether directly silencing postsynaptic EGNIS counteracted the behavioral effect of presynaptic inhibition. We investigated this by expressing Gi in the EGNIS (EGNIS^Gi^) of Aldh1a1^−/−^ mice, tdT (EGNIS^tdT^) was used as the control. We injected rAAV1/2-TH-DIO-TK/GFP virus and H129ΔTK-FLP virus into the VTA of Aldh1a1^−/−^-CRE mice, resulting in the expression of FLP in postsynaptic EGNIS. FLP recombination-dependent rAAV1/2-fDIO-Gi/tdT virus was injected into the IS, causing the expression of Gi in the Aldh1a1-targeted EGNIS (EGNIS^Gi^ mice, Fig. 4g). Mice were then administered with either saline or CNO (i.p., 5 mg kg^− 1^) 30 min before testing. All three groups, including EGNIS^Gi^-Aldh1a1^+/+^ mice given CNO and EGNIS^Gi^-Aldh1a1^−/−^ mice given CNO or saline, displayed similar values of C. S, R. T, and Accuracy in the touchscreen based reward choice tests (Supplementary Fig. 5d, e). Nevertheless, as compared with the controls, in which EGNIS^Gi^-Aldh1a1^−/−^ mice were treated with saline, EGNIS^Gi^-Aldh1a1^−/−^ mice given CNO exhibited normal delay of gratification; with the strong behavioral preference for LDR at day three of the probe trials (47.8 ± 1.2 trials% in Aldh1a1^+/+^-EGNIS^Gi^ mice given with CNO (blue), 52.6 ± 5.4 trials% in Aldh1a1^−/−^-EGNIS^Gi^ mice given with CNO (green) versus 13.2 ± 1.9 trials% in Aldh1a1^−/−^-EGNIS^Gi^ mice given with saline (red), mean ± SEM, *n* = 9 mice per group, Fig. 4b) and a high incidence of LRA visiting (68 ± 4.1% in Aldh1a1^+/+^-EGNIS^Gi^ mice given CNO, 66 ± 4.2% in Aldh1a1^−/−^-EGNIS^Gi^ mice given CNO versus 38.7 ± 3.1% in Aldh1a1^−/−^-EGNIS^Gi^ mice given saline, mean ± SEM, *n* = 11 mice per group, Fig. 4i). This finding reveals that direct silencing postsynaptic EGNIS shares the similarity with the presynaptic inhibition of Aldh1a1 neurons. Thus, Aldh1a1 → EGNIS synaptic transmission decodes delay of gratification and dysfunction of Aldh1a1 → EGNIS synaptic transmission causes impulsive behaviors.

### Aldh1a1 neurons receive excitatory synaptic inputs directly from L5PN

**Fig. 5:**
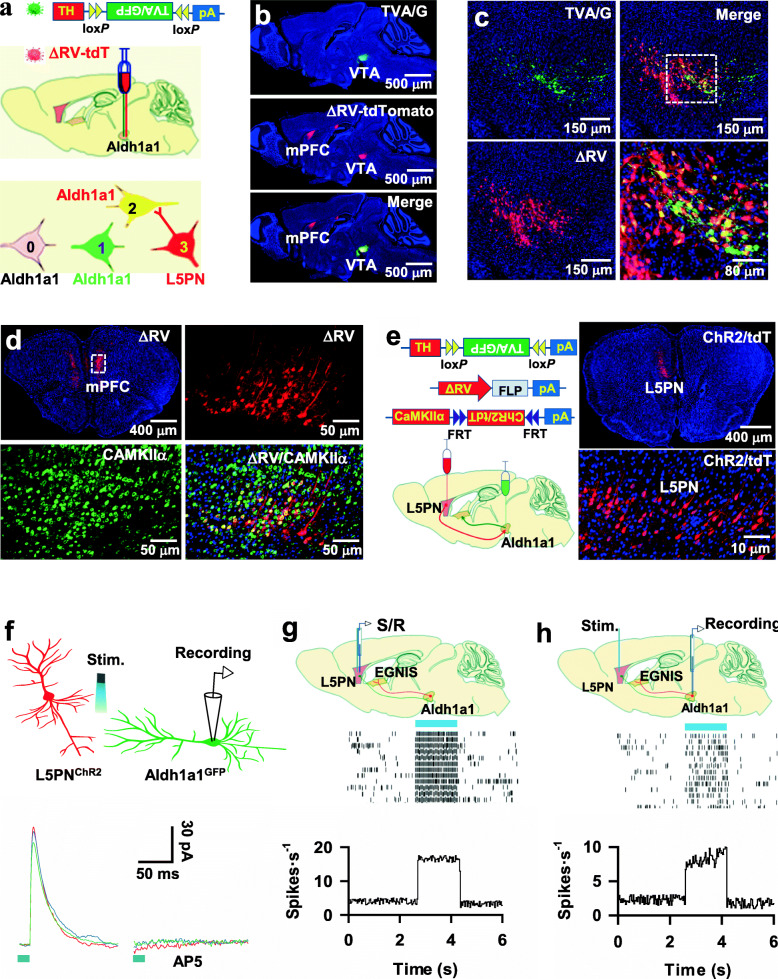
Genetic mapping synaptic inputs of Aldh1a1 neurons in adult mice. **a,** Illustration of retrograde tracing of synaptic inputs of Aldh1a1 neurons. Injection of rAAV1/2-TH-DIO-TVA/G virus causes the expression of TVA/G (green, 1) in CRE-expressing Aldh1a1 neurons (gray, 0). Twelve days later, ΔRV virus was injected, resulting in the expression of ΔRV in TVA/G-expressing Aldh1a1 neurons (yellow, 2) and neurons (red, 3) that project synapses directly onto Aldh1a1 neurons. **b,** The expression of ΔRV (red) in both the TVA/G-expressing Aldh1a1 neurons (green) and Aldh1a1 presynaptic neurons. **c,** The expression of ΔRV (red) in TVA/G-expressing Aldh1a1 neurons (yellow) in VTA. **d,** labeling of ΔRV-expressing neurons (red) with anti-CaMKIIα (green). **e,** Illustration (left) and the images (right) show the expression of ChR2 in L5PN. **f,** Three representative whole-cell patch-clamp recordings from Aldh1a1 neurons at a holding potential of + 60 mV. NMDA receptor-mediated EPSCs were evoked by delivery of blue laser light onto axons of ChR2-expressing L5PN and blocked by 100 μM AP5. **g, h**, The illustration (top) shows the recordings from L5PN (**g**) or Aldh1a1 neurons (**h**) of adult freely behaving mice. Raster plots (middle) show that delivery of blue laser light (horizontal bar) onto ChR2-expressing L5PN caused action potential firings in single L5PN (**g**) or Aldh1a1 neurons (**h**) of adult freely behaving mice. The summary plots (bottom) show that the delivery of blue laser light (horizontal bar) onto ChR2-expressing L5PN caused action potential firings in L5PN (*n* = 15 cell/5 mice) or Aldh1a1 neurons (*n* = 14 cells/5 mice) of adult freely behaving mice. All statistical data are summarized in Supplementary Table 1

To identify brain neurons that project their axon fibers directly onto Aldh1a1 neurons in the control of delayed gratification, we implemented retrograde synaptic mapping techniques by injecting the rAAV1/2-TH-DIO-TVA/G virus and synaptic retrograde ΔG-rabies viruses encoding tdT (ΔRV-tdT) into the VTA of Aldh1a1-CRE mice (Fig. 5a). This injection caused the expression of ΔRV-tdT in Aldh1a1 neurons (Fig. 5b, c) and their presynaptic neurons (Supplementary Fig. 6a), mainly located in layer 5b of the medial prefrontal cortex (L5, Fig. 5d, Supplementary Fig. 6b). Fluorescence labeling with an antibody against CaMKIIα revealed that ΔRV-labeled neurons were L5 excitatory pyramidal neurons (L5PN, Fig. 5d).

To determine a direct synaptic connection between L5PN and Aldh1a1 neurons, we engineered L5PN and Aldh1a1 neurons with the expression of ChR2/tdT and GFP, respectively. First, we expressed TVA/G and GFP in Aldh1a1 neurons by injecting the rAAV1/2-DIO-TVA/G-IRES-GFP virus in the VTA of Aldh1a1-CRE mice. Twelve days later, ΔRV-FLP virus was injected into the same area, resulting in the expression of FLP in L5PN. We applied FLP recombination-dependent rAAV1/2-fDIO-ChR2/tdT virus into the medial prefrontal cortex, causing the expression of ChR2/tdT specifically in L5PN (Fig. 5e). We performed whole-cell voltage-clamp recordings from GFP-expressing Aldh1a1 neurons in brain slices (Supplementary Fig. 7a). Blue laser light illumination of axon fibers of L5PN^ChR2/tdT^ with a brief pulse reliably evoked EPSCs, which were completely blocked by 20 μM CNQX (Supplementary Fig. 7b), showing an excitatory AMPA receptor-mediated synaptic response. To eliminate AMPA receptor-mediated polysynaptic effects, we recorded NMDA receptor-mediated EPSCs at a holding potential of + 60 mV from Aldh1a1 neurons in the presence of 20 μM CNQX (EPSCs-NMDA, Fig. 5f). EPSCs evoked by the stimulation of L5PN axon terminals were sensitive to NMDA receptor antagonist (2*R*)-amino-5-phosphonopentanoate (AP5) with a short latency, showing excitatory monosynaptic transmission (Fig. 5f, Supplementary Fig. 7c). Excitatory synaptic transmission from L5PN to EGNIS was also verified by demonstrating that blue laser light illumination of L5PN evoked action potential firing in both L5PN (Fig. 5g) and Aldh1a1 neurons (Fig. 5h) of freely behaving mice.

### Inhibition of L5PN → Aldh1a1 synaptic transmission induces impulsive behaviors

**Fig. 6:**
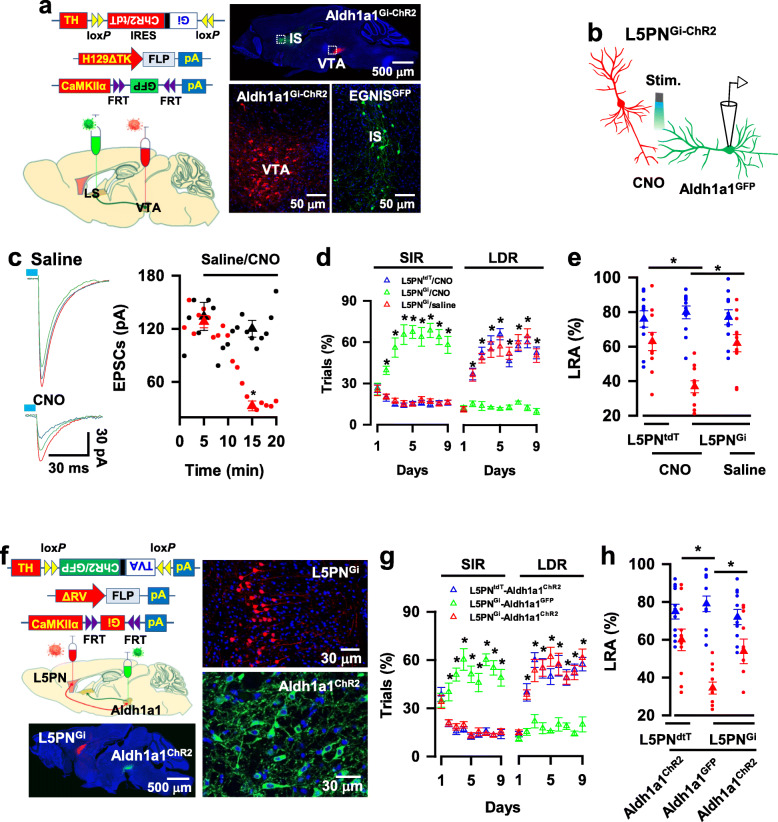
L5PN → Aldh1a1 synaptic transmission mediates delay of gratification. **a,** Representative images show the expression of Gi-ChR2 in L5PN (L5PN^Gi-ChR2^ mice). **b,** The illustration shows whole-cell patch-clamp recordings from GFP-expressing Aldh1a1 neurons and chemogenetic inhibition of L5PN axon terminals. **c,** EPSCs were recorded from GFP-expressing Aldh1a1 neurons and evoked by blue laser light stimulation on axon terminals of L5PN^Gi-ChR2^ in the slices. The plot shows the mean amplitudes of EPSCs versus the time of the individual (circles) recordings in the slices from L5PN^ChR2^ mice (black) or L5PN^Gi-ChR2^ mice (red) and the averages of the recordings 5 min from the baseline or the presence of CNO (mean ± SEM, *n* = 12 recordings/6 mice/group). **d**, Chemogenetic inhibition of L5PN outputs to Aldh1a1 neurons reduces the behavioral preference for LDR in the probe trials. The plot shows the percentage of the correct trials with the behavioral options for SIR or LDR of L5PN^tdT^ mice with CNO (blue) or L5PN^Gi^ mice with CNO (green) or saline (red) at each day of the probe trials (mean ± SEM, *n* = 9 mice per group). **e**, The chemogenetic inhibition of L5PN output to Aldh1a1 neurons reduces the percentage of LRA visiting. The plot shows the percentage of LRA visits at a delay of 0-3 s (blue) or 6-9 s (red) from individual (circles) L5PN^tdT^ mice with CNO or L5PN^Gi^ mice with CNO or saline and the averages per group (triangles, mean ± SEM, *n* = 11 mice/group) at day three of the testing sessions. **f**, Generation of a mutant line of mice with the expression of Gi in L5PN (L5PN^Gi^) and ChR2 in Aldh1a1 neurons (Aldh1a1^ChR2^). **g**, Optogenetic activation of Aldh1a1 neurons counteracts the effects of L5PN synaptic inhibition in delay of gratification. The plot shows the percentage of the correct trials with the behavioral options for SIR or LDR of L5PN^tdT^-Aldh1a1^ChR2^ (blue), L5PN^Gi^-Aldh1a1^GFP^ (green), or L5PN^Gi^-Aldh1a1^ChR2^ (red) mice at each day of the probe trials (mean ± SEM, *n* = 9 mice per group). In this study, CNO and blue laser light were delivered onto the VTA during the probe trials. **h**, The plot shows the percentage of LRA visits at a delay of 0-3 s (blue) or 6-9 s (red) from the individual (circles) L5PN^tdT^–Aldh1a1^ChR2^, L5PN^Gi^-Aldh1a1^GFP^, or L5PN^Gi^-Aldh1a1^ChR2^ mice and the averages per group (triangles, mean ± SEM, *n* = 11 mice per group) at day three of the testing sessions. All statistical data are summarized in Supplementary Table 1.

To determine the functionality of excitatory L5PN → Aldh1a1 synaptic transmission, we silenced the excitatory synaptic terminals of L5PN (Fig. 6a). A mutant line of mice expressing Gi-ChR2 in L5PN (L5PN^Gi-ChR2^) was generated by injecting rAAV1/2-DIO-TVA/G-IRES-GFP virus and the ΔRV-FLP virus into the VTA of Aldh1a1-CRE mice, resulting in the expression of TVA/G with GFP in Aldh1a1 neurons (Aldh1a1^GFP^) and FLP in L5PN (L5PN^FLP^). FLP recombination-dependent rAAV1/2-fDIO-Gi-ChR2/tdT virus was injected into the medial prefrontal cortex. This injection caused the expression of Gi-ChR2/tdT in L5PN (L5PN^Gi-ChR2^, Fig. 6a**).** Whole-cell patch-clamp recordings from Aldh1a1 neurons revealed that the application of CNO reduced the mean amplitude of EPSCs to 27 ± 2.1% of baseline (mean ± SEM, *n* = 12 recordings/6 mice, Fig. 6b and c), showing effective terminal inhibition. Subsequently, we examined the behavior 30 min after infusing CNO into the VTA. As shown in Fig. 6d, this terminal inhibition impaired delay of gratification. This resulted in a reduction in the behavioral preference for LDR in the probe trials (52.8 ± 4.3 trials% in L5PN^tdT^ mice given CNO (blue), 14 ± 3.2 trials% in L5PN^Gi^ mice given CNO (green) versus 49 ± 3.4 trials% in L5PN^Gi^ mice given saline (red) at day three of the probe trials, mean ± SEM, *n* = 9 mice per group), with decreased incidence of LRA visits in T-maze tests (63 ± 5.3% in L5PN^tdT^ mice given CNO, 36.8 ± 3.5% in L5PN^Gi^ mice given CNO versus 62 ± 5% in L5PN^Gi^ mice given saline, mean ± SEM, mean ± SEM, *n* = 11 mice per group, Fig. 6e). This impairment of the behavior was completely reversed by optogenetic activation of postsynaptic Aldh1a1 neurons (Fig. 6f). Optogenetic stimulation of Aldh1a1^ChR2^ neurons increased the behavioral preference of L5PN^Gi^ mice for LDR (60.6 ± 4.7 trials% in L5PN^tdT^-Aldh1a1^ChR2^ mice (blue), 18.5 ± 2 trials% in L5PN^Gi^-Aldh1a1^GFP^ mice (green) versus 51 ± 4 trials% in L5PN^Gi^-Aldh1a1^ChR2^ mice (red) at day three of the probe trials, mean ± SEM, *n* = 9 mice per group, Fig. 6g), without affecting the values of C. S, R. T, and Accuracy (Supplementary Fig. 7d, e). However, it significantly elevated the frequency of LRA visits (60 ± 5.7% in L5PN^tdT^- Aldh1a1^ChR2^ mice given with CNO, 34.5 ± 3.2% in L5PN^Gi^-Aldh1a1^GFP^ mice given with CNO versus 53.9 ± 6.5% in L5PN^Gi^-Aldh1a1^ChR2^ mice given with saline, mean ± SEM, mean ± SEM, *n* = 11 mice per group, Fig. 6h). Thus, L5PN → Aldh1a1 synaptic transmission decodes delay of gratification, and dysfunction of L5PN → Aldh1a1 synaptic transmission results in impulsive behaviors.

### Long-term potentiation (LTP) of L5PN → Aldh1a1 synaptic transmission decodes delay of gratification

**Fig. 7:**
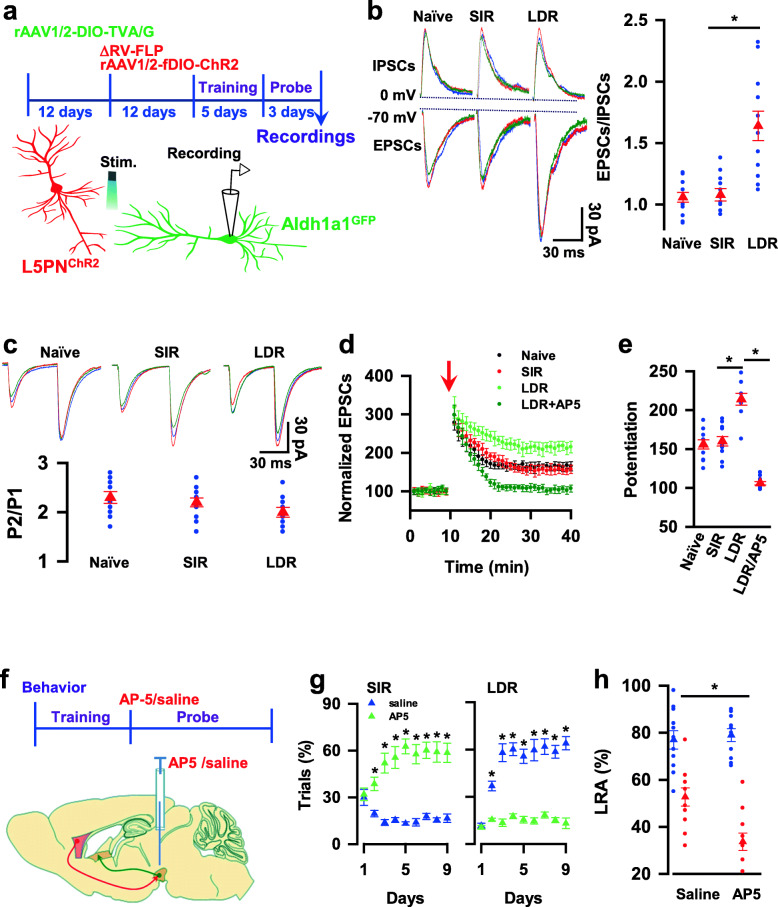
LTP of L5PN → Aldh1a1 synaptic transmission mediates delay of gratification. **a,** Experimental schedules for the generation of mutant mice with the expression of ChR2 in L5PN (L5PN^ChR2^) and GFP in Aldh1a1 neurons (Aldh1a1^GFP^) and whole-cell patch-clamp recordings of Aldh1a1^GFP^ neurons in the slices from mice after being tested. **b**, Delay of gratification enhances excitatory synaptic transmission from L5PN to Aldh1a1 neurons. IPSCs were recorded from Aldh1a1^GFP^ neurons at a holding potential of 0 mV and evoked by electrical stimulation on GABA inhibitory interneuron axon fibers. EPSCs were recorded from the same Aldh1a1^GFP^ neurons in the slices at a holding potential of −70 mV and evoked by blue laser light stimulation on L5PN^ChR2^ axon terminals. The plot shows the ratio of the mean amplitudes of EPSCs versus IPSCs from the individual recordings (blue circles) and the averages per group (red triangles, mean ± SEM, *n* = 12 recordings/6 mice/group). **c**, Paired-pulse facilitation was comparable among groups. EPSCs were recorded from Aldh1a1^GFP^ neurons and evoked by blue laser light stimulation on L5PN axon fibers with paired pulses at an interval of 50 ms. The ratio of pulse two versus pulse one (P2/P1) of the individual recordings (blue circles) and the averages per group (red triangles) was plotted. Data are the mean ± SEM (*n* = 12 recordings/6 mice/group). **d**, Delay of gratification potentiates synaptic transmission. The peak amplitudes of EPSCs in the slices from naïve, SIR, or LDR mice after being tested are normalized to the baseline (defined as 100) and plotted against the time of the recordings. The arrow indicates the time of tetanus, consisting of two trains of 100 Hz stimulation lasting 500 ms at an interval of 10 s. LDR/AP5 indicates that EPSCs are recorded in the slices from LDR mice in the presence of 100 μAP5. **e**, The normalized EPSCs during the last 5 min recordings (**d**) in the individual slices (blue circles) and the averages per group (mean ± SEM, *n* = 11 recordings/6 mice/group) are plotted. **f**, Experimental schedules show the infusion of 1 μl of 500 μM AP5 or saline into VTA 30 min before each day of the probe trials. **g**, Blocking L5PN → Aldh1a1 synaptic potentiation reduces the behavioral preference for LDR in the probe trials. The plot shows the percentage of the correct trials with the behavioral options for SIR or LDR of mice given AP5 (green) or saline (blue) at each day of the probe trials (mean ± SEM, *n* = 9 mice per group). **h**, Blocking L5PN → Aldh1a1 synaptic potentiation decreases the percentage of LRA visits. The plot shows the percentage of LRA visits with a delay of 0-3 s (blue) or 6-9 s (red) from the individual (circles) mice given AP5 or saline and the averages per group (triangles, mean ± SEM, *n* = 11 mice per group) at day three of the testing sessions. All statistical data are summarized in Supplementary Table 1

To explore whether a delay of gratification would modify the synaptic properties from L5PN to Aldh1a1 neurons, we generated L5PN^ChR2^-Aldh1a1^GFP^ mice, in which ChR2/tdT and GFP were expressed in L5PN and Aldh1a1 neurons, respectively. The VTA slices from L5PN^ChR2/tdT^-Aldh1a1^GFP^ mice without being tested (naïve mice) or with the behavioral preference (more than 55% trials) for LDR (LDR mice, *n* = 11) or more than 55% trials for SIR (SIR mice, *n* = 11) at the end of day three probe trials were prepared for ex vivo recordings of synaptic currents in Aldh1a1^GFP^ neurons (Fig. 7a). Blue laser light stimulation of L5PN axon terminals in the VTA generated a higher EPSC-AMPA to IPSC-GABA ratio in Aldh1a1 neurons from LDR mice, compared with SIR or naïve mice (1.06 ± 0.08 in naïve mice, 1.08 ± 0.05 in SIR mice versus 1.64 ± 0.12 in LDR mice, mean ± SEM, *n* = 12 recordings/6 mice/group, Fig. 7b). The mean amplitudes of IPSC-GABA (Supplementary Fig. 7f) and paired-pulse facilitation (PPF) of EPSC-AMPA were similar among groups (2.3 ± 0.12 in naïve mice, 2.2 ± 0.09 in SIR mice versus 2.0 ± 0.1 in LDR mice, mean ± SEM, *n* = 12 recordings/6 mice/group, Fig. 7c). Next, we recorded LTP of excitatory synaptic transmission, which is a major form of synaptic plasticity considered as a cellular substrate of value-directed decision making. We observed that LTP of EPSCs-AMPA was significantly higher in LDR mice than in the other groups (156 ± 5.9 in naïve, 160 ± 6.1 in SIR versus 214 ± 7.6 in LDR and 106 ± 2.2 in LDR + AP5, mean ± SEM, *n* = 11 recordings/6 mice/group, Fig. 7d, e), and this potentiation was completely blocked by the NMDA receptor antagonist AP5, confirming a conventional NMDA receptor-dependent mechanism for LTP induction. Thus, delay of gratification potentiates synaptic strength in postsynaptic Aldh1a1 neurons. To test for causality, we applied NMDA receptor antagonist 1 μl of 500 μM AP5 into the VTA on each day of the probe trials (Fig. 7f). This application decreased the behavioral option for LDR in the probe trials (58 ± 5.7 trials% in saline (blue) versus 13.6 ± 2.3 trials% in AP5 (green) in day three, mean ± SEM, mean ± SEM, *n* = 9 mice per group, Fig. 7g), produced no change in C. S, R. T, and Accuracy (Supplementary Fig. 7 g, h) and significantly reduced the frequency of LRA visiting (52.7 ± 3.9% in saline versus 33.7 ± 3.7% in AP5, mean ± SEM, *n* = 11 mice per group, Fig. 7h). Thus, delay of gratification depends on a long-lasting enhancement of L5PN → Aldh1a1 synaptic transmission.

Impulsive behavior occurs at the early stage of AD [39, 40]. Subsequently, we examined Aldh1a1 → EGNIS synaptic transmission in heterozygous APPswe/PSEN1dE9 mutant mice (AD mice) carrying a transgene encoding the 695-amino-acid isoform of the human Aβ precursor protein with the Swedish mutation and a mutant human presenilin1 (PS1-dE9), which displayed impulsive behaviors when they were 5 months old, as compared with non-transgenic control C57BL/6 mice (Supplementary Fig. 8a). AD mice at 5 months old showed a reduction in Aldh1a1 expression in Aldh1a1 neurons (Supplementary Fig. 8b) and a dysfunction of Aldh1a1 → EGNIS synaptic transmission (Supplementary Fig. 8c). Introduction of eAldh1a1 in Aldh1a1 neurons restored Aldh1a1 → EGNIS synaptic transmission and rescued the impulsive behaviors (Supplementary Fig. 1d-f), demonstrating that dysfunction of Aldh1a1 → EGNIS synaptic transmission contributes to the impairment of delayed gratification in AD.

## Discussion

This study has applied genetically modified retrograde and anterograde synaptic tracing approach and carried out an integrative study genetically linking a synaptic circuit of Aldh1a1 neurons to its systems-level function and pathological relevance. We have reported a discovery that Aldh1a1 neurons decode delay of gratification by synapsing directly with EGNIS, and synaptic dysfunction of Aldh1a1 neurons causes impulsive behavior. Thus, this study has not only highlighted a behavioral function and input-output synaptic connectivity of Aldh1a1 neurons but also pinpoints a cellular point of entry to an understudied pathological node that mediates impulsive behaviors in AD (Supplementary Fig. 9).

Delay of gratification is the psychological process that underlies decisions involving outcomes at different points over time and relies largely on cognitive control skills, executive functions, and value-directed decision making [41–45]. It has been studied extensively in a classical psychological experiment known as the Stanford marshmallow test [34, 46, 47], in which children were asked to choose a single marshmallow now or two in 15 min. The individuals who chose to wait went on to do better at school and show greater social, economic, and academic success in later life than those who ate a single marshmallow [34, 43, 47–54]. Cognitive control skills reflect the ability to suppress competing inappropriate thoughts or actions in favor of appropriate ones [55, 56]. Previous studies indicated that the capacity to delay gratification in childhood predicts the efficiency with which the same individuals perform a cognitive control task as adolescents and young adults [34, 47–50, 52, 53]. Functional magnetic resonance imaging (fMRI) and lesion studies in both human and non-human primates have indicated that both the medial prefrontal cortex and the dorsal raphe nucleus are involved in cognitive control during the delay of rewards, whereas limbic regions are associated with impulsive behaviors [31–34, 36, 37]. However, due to the lack of techniques for selectively labeling, mapping, and screening of a specific type of neurons in the adult brain, which of over hundreds of thousands of neurons in each of these brain regions specifically encode delay of gratification remains unknown. In this study, we have genetically targeted Aldh1a1 neurons in the ventral tegmental area of adult mice. We have also developed two independent strategies to genetically manipulate individual Aldh1a1 neurons and their circuitry in freely behaving adult and AD mice. We have provided a synaptic and circuit mechanism for encoding delay of gratification.

In this study, we have deleted Aldh1a1 gene in the individual Aldh1a1 neurons (Aldh1a1^−/−^ mice) and carried out two independent behavioral tests: a touchscreen-based behavioral options for rewards that varied in both sizes and delays, and T-maze tests for delay-based decision making. In touchscreen-based tests, Aldh1a1^−/−^ mice show the behavioral options for a small immediate reward rather than a large delayed reward. This behavioral option differs from the wildtype control mice, which prefer to a large delayed reward instead of a small immediate reward. One interpretation is that Aldh1a1^−/−^ mice are less sensitive to reward magnitude and therefore don’t value the difference in reward sizes, and hence gravitate to a more immediate option. We have excluded this possibility by T-maze tests, in which mice have a choice between a small and a large reward with the same short delay (0-3 s). We have found that Aldh1a1^−/−^ mice display no difference from wildtype control mice with the preference for a large reward (Fig. 2h). It is also possibly that Aldh1a1^−/−^ mice are more sensitive to delay than control mice. To validate this possibility, we have assessed the behavioral options for a small reward with short delay (0-3 s) versus a large reward with the long delay (6-9 s) and found that Aldh1a1^−/−^ mice choose a small reward instead of a large one (Fig. 2h). Thus, we conclude that Aldh1a1^−/−^ mice are impaired in delay of gratification.

Choosing a small reward now or a large one later involves several psychological and pathological processes. Before choosing, individuals need to use their previous experience to compare the value of the immediate versus the delayed rewards. Individuals who are hungry may assign a greater value to eating a single marshmallow now than those who feel full. After making a choice, the individuals must estimate whether a received reward is their expected reward and therefore adjust their behavioral options for the future. This behavioral option for delayed reward has been studied mainly in human and non-human primates. Our present study has demonstrated that genetic manipulations of Aldh1a1 neurons and their circuits in adult mice altered the behavioral preference for a large delayed reward, without affecting the accuracy or the correct scores (C.S) and reaction times (R.T), as the measures of reward learning and motivation. Thus, Aldh1a1 neurons function as a cellular substrate for delay of gratification via innervation of neurons in the intermediate lateral septum.

In this study, we have also assessed the behaviors and synaptic functions of Aldh1a1 neurons in AD mice. Our data have revealed that synapses of Aldh1a1 neurons are degenerated and associated with the impairments of the behavioral options for a large delayed reward. This finding supports the previous reports that impulsive behaviors occur in the early stage of human patients with AD [39, 40]. Notably, an early study indicates that AD patients displayed significantly difference from control group in the degree of impulsive behaviors at one-month or one-year delay assay, but they had no difference from control group in the degree of impulsive behaviors at ten-years delay measurements [57]. This negative finding could be due to the participants (averaging over 70 years old) may consider that he or she won’t be in a sufficient physical condition to get the reward in 10 years, considering that the rewards after ten-years delay involved long journeys (i.e., to watch the tennis game in France or visit the Great Wall of China.).

## Conclusions

In conclusion, we performed an integrative study using retrograde and anterograde synaptic tracing methods linking a specific synaptic and circuitry mechanism with the systems-level function of Aldh1a1 neurons. We discovered a specific function and circuit of Aldh1a1 neuron decoding delay of gratification and provided a cellular point of entry to a previously unrecognized synaptic node in the brain circuitry for control of impulsive behaviors (Supplementary Fig. 9). The high capacity to delay gratification predicts social, economic, and academic success, whereas behavioral preference for a small, more immediate reward over a large delayed reward is a hallmark of attention deficit hyperactivity disorders, stress, and drug abuse [31, 58]. Thus, our finding of Aldh1a1 neurons in the control of impulsive behaviors warrants a specific cellular target for the therapeutic intervention of value-directed decision making diseases such as AD.

## Supplementary Information

**Additional file 7.**

**Additional file 8: Supplementary Fig. 1.** Genetic labeling and tracing of Aldh1a1 neurons and their synaptic targets. **Supplementary Fig. 2**. Aldh1a1 neurons release GABA and DA transmitters. **Supplementary Fig. 3**. Aldh1a1-/- mice show normal motor activity. **Supplementary Fig. 4**. Deletion of Aldh1a1 produces no effects on motivation for reward. **Supplementary Fig. 5** Chemogenetic silencing synaptic transmission^Aldh1a1→EGNIS^. **Supplementary Fig. 6**. Expression of RV in presynaptic neurons of Aldh1a1 neurons. **Supplementary Fig. 7**. Glutamate excitatory synaptic transmission^L5PN→ Aldh1a1^. **Supplementary Fig. 8**. Dysfunction of Aldh1a1→EGNIS synaptic transmission causes impulsive behaviors in AD mice. **Supplementary Fig. 9**. A novel circuitry of Aldh1a1 neurons encodes delay of gratification. **Supplementary Fig. 10**. Vector design and genotype for generation of Aldh1a1-CRE mice. **Supplementary Fig. 11** a, b, The behavioral tests comprised of training session (a) and probe trials (b). In the training sessions, each trial was started when a house light on. After 3 s, one of the three cue symbols was displayed on the touchscreen for 5 s. Mice were required to nose-poking this symbol within 5 s and collected a contingency reward with a specific delay. After successfully trained (>75% accuracy), mice were subjected to the probe trials, in which mice were required to freely choose among three symbols that were displayed on the touchscreen for 5 s.

## Data Availability

All data generated and/or analyzed during this study are included in the article and the Source Data files. Any additional information required is available from the corresponding author on reasonable request.

## Abbreviations

Aldh1a1: Aldehyde dehydrogenase 1a1
VTA: Ventral tegmental area
EGNIS: Excitatory glutamate neurons in the intermediate lateral septum
L5PN: Layer 5b pyramidal neurons in the medial prefrontal cortex
SIR: Small immediate available reward
LDR: Large delayed reward
LLR: Largest long delayed reward
LTP: Long-term potentiation
