# Correction: Synaptic dysfunction of Aldh1a1 neurons in the ventral tegmental area causes impulsive behaviors

**DOI:** 10.1186/s13024-023-00607-6

**Published:** 2023-05-15

**Authors:** Xinyan Li, Wenting Chen, Xian Huang, Wei Jing, Tongmei Zhang, Quntao Yu, Hongyan Yu, Hao Li, Qing Tian, Yumei Ding, Youming Lu

**Affiliations:** 1 Department of Physiology, School of Basic Medicine and Tongji Medical College, Huazhong University of Science and Technology, Wuhan, 4030030 China; 2 Wuhan Center of Brain Science, Huazhong University of Science and Technology, Wuhan, 430030 China; 3 Department of Neurobiology, School of Basic Medicine and Tongji Medical College, Huazhong University of Science and Technology, Wuhan, 430030 China; 4 Department of Pathophysiology, School of Basic Medicine and Tongji Medical College, Huazhong University of Science and Technology, Wuhan, 430030 China; 5 Department of Stomatology, School of Stomatology, Union Hospital, Tongji Medical College, Huazhong University of Science and Technology, Wuhan, 430030 China

**Correction: Mol Neurodegeneration 16, 73 (2021)**

**https://doi.org/10.1186/s13024-021-00494-9**

In the version initially published online of this article [1], the representative images of Fig. 4a and Fig. 6a were misused. An image of Aldh1a1^Gi-ChR2^ in Fig. 4a (lower left) and an image of L5PN^Gi-ChR2^ in Fig. 6a (right) were the photographs from the other respective experimental groups. The errors have been corrected in PDF version of the article. This correction has been appended to the PDF version. The authors regret the errors.

**Fig. 4:**
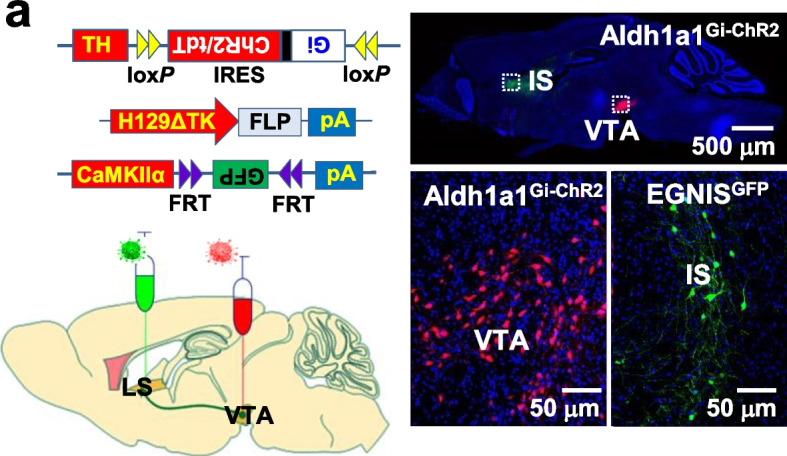
**a**, The expression of Gi-ChR2 and GFP in Aldh1a1 neurons (Aldh1a1^Gi-ChR2^) and EGNIS (EGNIS^GFP^), respectively

**Fig. 6:**
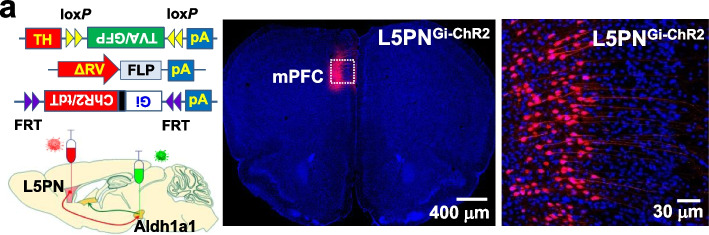
**a**, Representative images show the expression of Gi-ChR2 in L5PN (L5PN^Gi-ChR2^ mice)
