# Supplementary material for: Synaptic dysfunction of Aldh1a1 neurons in the ventral tegmental area causes impulsive behaviors
Source: Mol Neurodegener. 2021 Oct 26;16:73. doi: 10.1186/s13024-021-00494-9 (PMC8549305; doi:10.1186/s13024-021-00494-9)
Supplement: Supplementary file 7 — Additional file 7. [file 13024_2021_494_MOESM7_ESM.docx]

**Supplementary Table 1.**

**Statistics about *t*-test**

| Figure | n | p | t |
| --- | --- | --- | --- |
| 2c | 5 | 0.0006 | 5.415 |
| 3c-Aldh1a1^+/+^ | 12 | 0.9046 | 0.1213 |
| 3c-Aldh1a1^-/-^ | 12 | <0.0001 | 21.33 |
| S3a-light | 7 | 0.5806 | 0.5678 |
| S3a-dark | 7 | 0.7638 | 0.3074 |
| S3b | 7 | 0.8078 | 0.2487 |
| S3c | 7 | 0.6985 | 0.3968 |
| S3d-open-arm | 7 | 0.6627 | 0.4472 |
| S3d-time | 7 | 0.8406 | 0.205 |
| S3e-training | 7 | 0.09962 | 0.9223 |
| S3e-testing | 7 | 0.6532 | 0.4608 |
| S5a | 11 | <0.0001 | 8.955 |
| S8b | 5 | 0.006 | 3.7 |
| S8c | 9 | <0.0001 | 10.47 |

**Statistics about one-way *ANOVA***

| Figure | n | F | P |
| --- | --- | --- | --- |
| 2d | 7 | (2,18)=19.35 | <0.0001 |
| 7b | 12 | (2, 33) = 18.04 | <0.0001 |
| 7c | 12 | (2, 33) = 1.326 | 0.2794 |
| 7e | 11 | (3, 40) = 58.9 | <0.0001 |
| S7f | 12 | (2, 33) = 0.01973 | 0.9805 |
| S8e | 9 | (2, 24) = 43.67 | <0.0001 |

**Statistics about two-way *ANOVA***

| Figure | ***ANOVA* table** | n | F | P |
| --- | --- | --- | --- | --- |
| 2f-SIR | Group | 9 | (1, 144)=186.3 | <0.0001 |
|  | Day |  | (8, 144)=2.396 | 0.0185 |
|  | Group×Day |  | (8, 144)=5.544 | <0.0001 |
| 2f-LDR | Group | 9 | (1, 144) = 182.7 | <0.0001 |
|  | Day |  | (8, 144) = 5.675 | <0.0001 |
|  | Group×Day |  | (8, 144) = 4.266 | 0.0001 |
| 2f-LLR | Group | 9 | (1, 144) = 0.9433 | 0.3331 |
|  | Day |  | (8, 144) = 1.286 | 0.2554 |
|  | Group×Day |  | (8, 144) = 0.2135 | 0.9197 |
| 2h-0-3s | Group | 11 | (1, 64) = 0.07401 | 0.9287 |
|  | Day |  | (2, 64) = 0.3158 | 0.5763 |
|  | Group×Day |  | (2, 64) = 9.606 | 0.0002 |
| 2h-6-9s | Group | 11 | (1, 64) = 31.16 | <0.0001 |
|  | Day |  | (2, 64) = 2.546 | 0.0868 |
|  | Group×Day |  | (2, 64) = 3.232 | 0.0465 |
| 3d-C.S | Group | 9 | (3, 288) = 0.8172 | 0.4852 |
|  | Day |  | (8, 288) = 25.2 | <0.0001 |
|  | Group×Day |  | (24, 288) = 0.22 | >0.9999 |
| 3e-R.T | Group | 9 | (3, 288) = 0.3066 | 0.8206 |
|  | Day |  | (8, 288) = 14.31 | <0.0001 |
|  | Group×Day |  | (24, 288) = 0.44126 | 0.9941 |
| 3f-SIR | Group | 9 | (3, 288) = 164 | <0.0001 |
|  | Day |  | (8, 288) = 1.187 | 0.3064 |
|  | Group×Day |  | (24, 288) = 3.751 | <0.0001 |
| 3f-LDR | Group | 9 | (3, 288) = 78.07 | <0.0001 |
|  | Day |  | (8, 288) = 20.78 | <0.0001 |
|  | Group×Day |  | (24, 288) = 1.65 | 0.0309 |
| 3f-LLR | Group | 9 | (3, 288) = 0.454 | 0.7147 |
|  | Day |  | (8, 288) = 3.826 | 0.0003 |
|  | Group×Day |  | (24, 288) = 0.1401 | >0.9999 |
| 3g-Aldh1a1^+/+^ | Group | 11 | (1, 40) = 0.02528 | 0.8745 |
|  | Test |  | (1, 40) = 14.73 | 0.0004 |
|  | Group×Test |  | (1, 40) = 0.4643 | 0.4996 |
| 3g-Aldh1a1^-/-^ | Group | 11 | (1, 40) = 23.10 | <0.0001 |
|  | Test |  | (1, 40) = 56.42 | <0.0001 |
|  | Group×Test |  | (1, 40) = 11.27 | 0.0017 |
| 4e-SIR | Group | 9 | (2, 216) = 193 | <0.0001 |
|  | Day |  | (8, 216) = 0.4233 | 0.9063 |
|  | Group×Day |  | (16, 216) = 4.82 | <0.0001 |
| 4e-LDR | Group | 9 | (2, 216) = 127.4 | <0.0001 |
|  | Day |  | (8, 216) = 13.78 | <0.0001 |
|  | Group×Day |  | (16, 216) = 2.929 | 0.0002 |
| 4e-LLR | Group | 9 | (2, 216) = 0.4628 | 0.6302 |
|  | Day |  | (8, 216) = 4.586 | <0.0001 |
|  | Group×Day |  | (16, 216) = 0.8492 | 0.6285 |
| 4f | Group | 11 | (1, 60) = 48.66 | <0.0001 |
|  | Test |  | (2, 60) = 9.554 | 0.0003 |
|  | Group×Test |  | (2, 60) = 5.269 | 0.0078 |
| 4h-SIR | Group | 9 | (2, 216) = 215.6 | <0.0001 |
|  | Day |  | (8, 216) = 0.8768 | 0.5369 |
|  | Group×Day |  | (16, 216) = 4.523 | <0.0001 |
| 4h-LDR | Group | 9 | (2, 216) = 204.8 | <0.0001 |
|  | Day |  | (8, 216) = 14.15 | <0.0001 |
|  | Group×Day |  | (16, 216) = 4.703 | <0.0001 |
| 4i | Group | 11 | (1, 60) = 25.28 | <0.0001 |
|  | Test |  | (2, 60) = 6.961 | 0.0019 |
|  | Group×Test |  | (2, 60) = 10.87 | <0.0001 |
| 6d-SIR | Group | 9 | (2, 270) = 339.2 | <0.0001 |
|  | Day |  | (8, 270) = 1.627 | 0.1185 |
|  | Group×Day |  | (16, 270) = 7.843 | <0.0001 |
| 6d-LDR | Group | 9 | (2, 270) = 257.4 | <0.0001 |
|  | Day |  | (8, 270) = 22.91 | <0.0001 |
|  | Group×Day |  | (16, 270) = 6.235 | <0.0001 |
| 6e | Group | 11 | (1, 60) = 42.02 | <0.0001 |
|  | Test |  | (2, 60) = 4.231 | 0.0191 |
|  | Group×Test |  | (2, 60) = 6.545 | 0.0027 |
| 6g-SIR | Group | 9 | (2, 270) = 253.8 | <0.0001 |
|  | Day |  | (8, 270) = 3.167 | 0.0021 |
|  | Group×Day |  | (16, 270) = 6.049 | <0.0001 |
| 6g-LDR | Group | 9 | (2, 270) = 156.8 | <0.0001 |
|  | Day |  | (8, 270) = 16.87 | <0.0001 |
|  | Group×Day |  | (16, 270) = 2.764 | 0.0005 |
| 6h | Group | 11 | (1, 60) = 45.17 | <0.0001 |
|  | Test |  | (2, 60) = 2.508 | 0.0899 |
|  | Group×Test |  | (2, 60) = 5.984 | 0.0043 |
| 7g-SIR | Group | 9 | (1, 144) = 197.1 | <0.0001 |
|  | Day |  | (8, 144) = 1.197 | 0.3047 |
|  | Group×Day |  | (8, 144) = 5.979 | <0.0001 |
| 7g-LDR | Group | 9 | (1, 144) = 496.2 | <0.0001 |
|  | Day |  | (8, 144) = 13.93 | <0.0001 |
|  | Group×Day |  | (8, 144) = 9.932 | <0.0001 |
| 7h | Group | 11 | (1, 40) = 85.17 | <0.0001 |
|  | Test |  | (1, 40) = 9.623 | 0.0035 |
|  | Group×Test |  | (1, 40) = 13.65 | 0.0007 |
| S2b | Transmitter | 5 | (2,60)=23.53 | <0.0001 |
|  | Time |  | (4,60)=70.91 | <0.0001 |
|  | Transmitter×Time |  | (8,60)=20.12 | <0.0001 |
| S2c | Group | 5 | (1,40)=0.06308 | 0.803 |
|  | Time |  | (4,40)=74.32 | <0.0001 |
|  | Group×Time |  | (4,40)=0.148 | 0.9628 |
| S2d | Group | 5 | (1,40)=23.27 | <0.0001 |
|  | Time |  | (4,40)=33.54 | <0.0001 |
|  | Group×Time |  | (4,40)=24.76 | <0.0001 |
| S4a-C.S | Group | 9 | (1, 144)=0.0004346 | 0.9834 |
|  | Day |  | (8, 144)=9.801 | <0.0001 |
|  | Group×Day |  | (8, 144)=0.661 | 0.7251 |
| S4a-R.T | Group | 9 | (1, 144)=1.207 | 0.2738 |
|  | Day |  | (8, 144)=10.55 | <0.0001 |
|  | Group×Day |  | (8, 144)=0.2257 | 0.9858 |
| S4c | Group | 15 | (1, 140)=0.001067 | 0.974 |
|  | Day |  | (4, 140)=59.84 | <0.0001 |
|  | Group×Day |  | (4, 140)=0.583 | 0.6755 |
| S5b-C.S | Group | 9 | (2, 216)=4.747 | 0.0096 |
|  | Day |  | (8, 216)=23.99 | <0.0001 |
|  | Group×Day |  | (16, 216)=1.011 | 0.4464 |
| S5b-R.T | Group | 9 | (2, 216)=17.48 | 0.6784 |
|  | Day |  | (8, 216)=31.24 | <0.0001 |
|  | Group×Day |  | (16, 216)=0.479 | 0.9553 |
| S5d-C.S | Group | 9 | (2, 216)=0.8153 | 0.4439 |
|  | Day |  | (8, 216)=44.07 | <0.0001 |
|  | Group×Day |  | (16, 216)=0.4206 | 0.9762 |
| S5d-R.T | Group | 9 | (2, 216)=0.09145 | 0.9126 |
|  | Day |  | (8, 216)=9.767 | <0.0001 |
|  | Group×Day |  | (16, 216)=0.4802 | 0.9548 |
| S7d-C.S | Group | 9 | (2, 270)=0.457 | 0.6337 |
|  | Day |  | (8, 270)=36.9 | <0.0001 |
|  | Group×Day |  | (16, 270)=0.7636 | 0.7261 |
| S7d-R.T | Group | 9 | (2, 270)=0.08997 | 0.914 |
|  | Day |  | (8, 270)=21.67 | <0.0001 |
|  | Group×Day |  | (16, 270)=0.9515 | 0.5107 |
| S7g-C.S | Group | 9 | (1,144)=0.3155 | 0.5752 |
|  | Day |  | (8,144)=24.8 | <0.0001 |
|  | Group×Day |  | (8,144)=0.1047 | 0.999 |
| S7g-R.T | Group | 9 | (1,144)=0.166 | 0.6843 |
|  | Day |  | (8,144)=9.153 | <0.0001 |
|  | Group×Day |  | (8,144)=0.983 | 0.4518 |
| S8a-SIR | Group | 9 | (1,144)=110 | <0.0001 |
|  | Day |  | (8,144)=0.1898 | 0.9920 |
|  | Group×Day |  | (8,144)=3.509 | 0.001 |
| S8a-LDR | Group | 9 | (1,144)=168.2 | <0.0001 |
|  | Day |  | (8,144)=15.3 | <0.0001 |
|  | Group×Day |  | (8,144)=2.961 | 0.0043 |
| S8f-SIR | Group | 9 | (2, 216)=104.8 | <0.0001 |
|  | Day |  | (8, 216)=0.9292 | 0.4931 |
|  | Group×Day |  | (16, 216)=2.968 | 0.0002 |
| S8f-LDR | Group | 9 | (2, 216)=71 | <0.0001 |
|  | Day |  | (8, 216)=15.06 | <0.0001 |
|  | Group×Day |  | (16, 216)=1.013 | 0.4445 |
