# Supplementary material for: Synaptic dysfunction of Aldh1a1 neurons in the ventral tegmental area causes impulsive behaviors
Source: Mol Neurodegener. 2021 Oct 26;16:73. doi: 10.1186/s13024-021-00494-9 (PMC8549305; doi:10.1186/s13024-021-00494-9)
Supplement: Supplementary file 8 — Additional file 8: Supplementary Fig. 1. Genetic labeling and tracing of Aldh1a1 neurons and their synaptic targets. Supplementary Fig. 2. Aldh1a1 neurons release GABA and DA transmitters. Supplementary Fig. 3. Aldh1a1-/- mice show normal motor activity. Supplementary Fig. 4. Deletion of Aldh1a1 produces no effects on motivation for reward. Supplementary Fig. 5 Chemogenetic silencing synaptic transmissionAldh1a1→EGNIS. Supplementary Fig. 6. Expression of RV in presynaptic neurons of Aldh1a1 neurons. Supplementary Fig. 7. Glutamate excitatory synaptic transmissionL5PN→ Aldh1a1. Supplementary Fig. 8. Dysfunction of Aldh1a1→EGNIS synaptic transmission causes impulsive behaviors in AD mice. Supplementary Fig. 9. A novel circuitry of Aldh1a1 neurons encodes delay of gratification. Supplementary Fig. 10. Vector design and genotype for generation of Aldh1a1-CRE mice. Supplementary Fig. 11 a, b, The behavioral tests comprised of training session (a) and probe trials (b). In the training sessions, each trial was started when a house light on. After 3 s, one of the three cue symbols was displayed on the touchscreen for 5 s. Mice were required to nose-poking this symbol within 5 s and collected a contingency reward with a specific delay. After successfully trained (>75% accuracy), mice were subjected to the probe trials, in which mice were required to freely choose among three symbols that were displayed on the touchscreen for 5 s. [file 13024_2021_494_MOESM8_ESM.pdf]

Supplementary Fig. 1

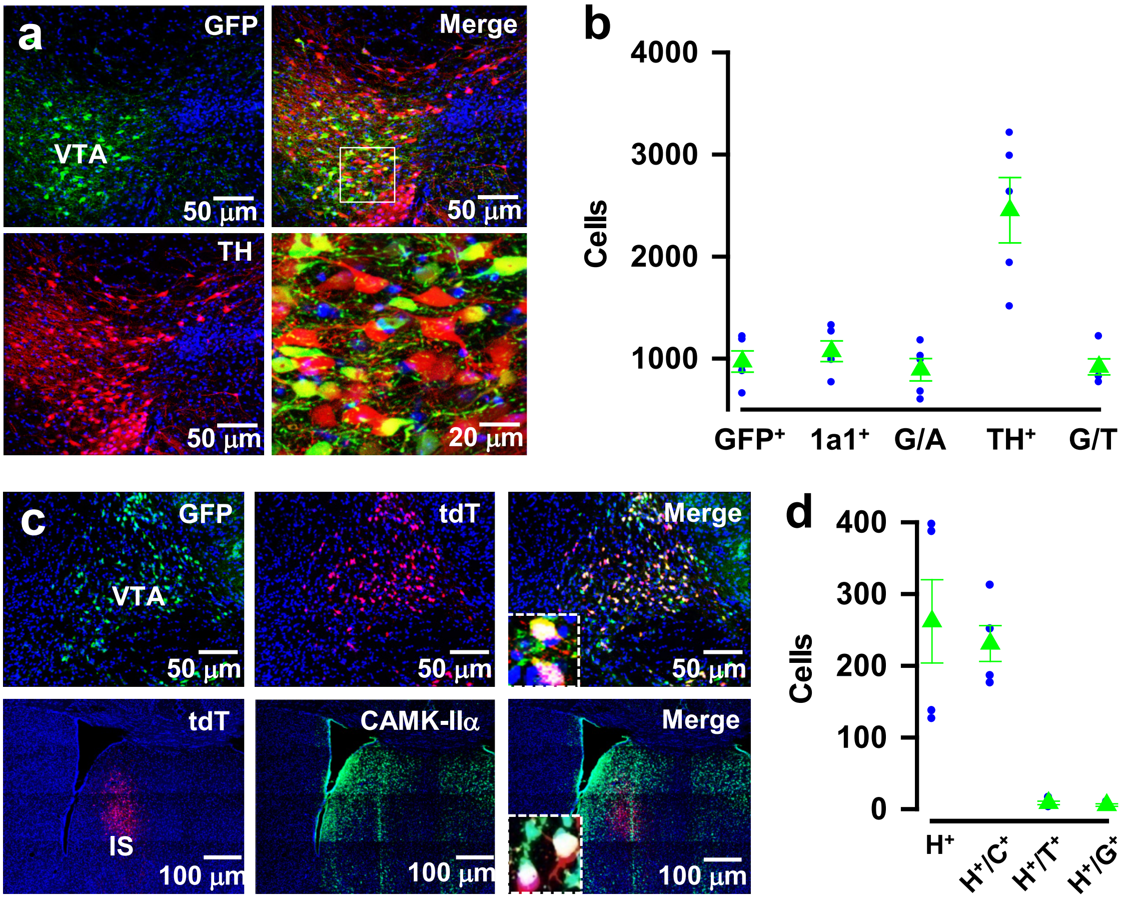

Supplementary Fig. 1. Genetic labeling and tracing of Aldh1a1 neurons and their synaptic targets. a, Representative images show the labeling of GFP-expressing Aldh1a1 neurons with anti-TH (also see Figure 1C). **b,** The rAAV1/2-TH-DIO-GFP virus was injected into VTA of Aldh1a1-CRE mice. The brain sections were then stained with anti-Aldh1a1 ($1a1^+$) or anti-TH (TH^+) antibodies. The number of GFP-expressing neurons (GFP^+), GFP^+/Aldh1a1$^+$ neurons (G/A), TH^+ neurons, and GFP^+/TH^+ neurons (G/T) was counted and plotted from the individual mice (blue circles) and their averages per group (green triangles, mean ± SEM, n = 5). **c,** Representative images (top) show the labeling of GFP-expressing Aldh1a1 neurons with the expression of H129 after the injection of the rAAV1/2-TH-DIO-TK virus together with the H129ΔTK virus into VTA of Aldh1a1-CRE mice (also see Figure 1G). The images (bottom) show the labeling of tdT-expressing neurons in IS with anti-CAMKIIα (also see Figure 1H). **d,** Labeling of tdT (H)-expressing neurons in IS with anti-CAMKIIα(C^+), anti-CHAT (T^+) or anti-GAD67 (G^+) antibodies. A plot shows the number of H^+, H^+/C^+, H^+/T^+ and H^+/G^+ cells from the individual mice (blue circles) and their averages per group (green triangles, mean ± SEM, n = 5).

Supplementary Fig. 2

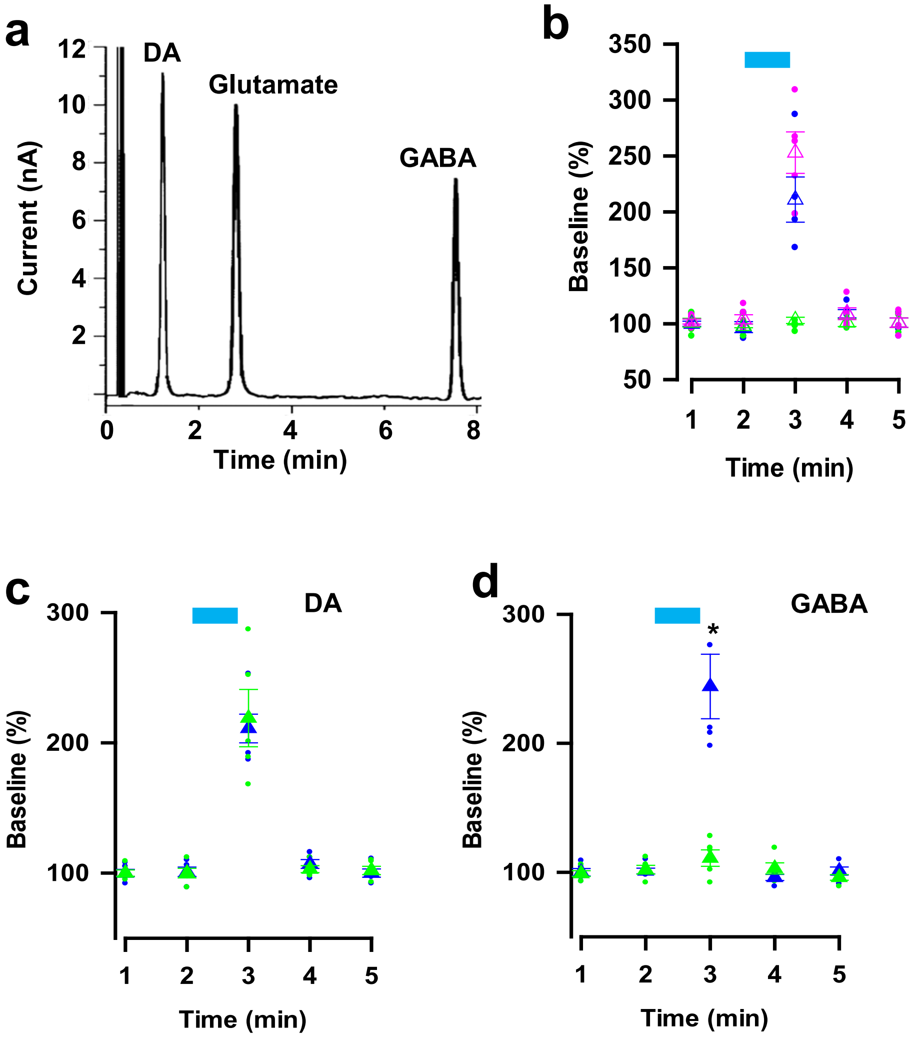

Supplementary Fig. 2. Aldh1a1 neurons release GABA and DA transmitters. a, Analysis of 2 μL injection of a mixture of DA (2 μM), glutamate (1 μM) and GABA (1 μM). **b,** Activation of Aldh1a1 neurons increases GABA and DA transmitter release. The rAAV1/2-TH-DIO-ChR2 virus was injected into VTA of Aldh1a1-CRE, resulting in the expression of ChR2 in Aldh1a1 neurons. Transmitters in the dialysis samples from IS were measured using high performance liquid chromatography (HPLC) with fluorescence detection (HPLC-FD). Blue laser lights were delivered onto ChR2-expressing Aldh1a1 neurons (horizontal bar) after 2-min baseline (defined as 100%). A plot shows the normalized levels of glutamate (green) and GABA (red) and DA (blue) from the individual mice (circles) and the averages per group (triangles, mean ± SEM, n = 5 mice per group; two-way *ANOVA*, Transmitter × Time $F_{(8, 60)} = 20.12$, $p < 0.0001$, Transmitter $F_{(2, 60)} = 23.53$, $p < 0.0001$, Time $F_{(4, 60)} = 70.91$, $p < 0.0001$, Bonferroni *post-hoc*). **c,** Deletion of Aldh1a1 produces no affect on DA release from Aldh1a1 neurons. Blue laser lights were delivered (horizontal bar) after 2-min baseline. A plot shows the normalized levels of DA to the baseline (100%) from the individual (circles) Aldh1a1$^{+/+}$ (blue) and Aldh1a1$^{-/-}$ (green) mice and the averages per group (triangles, mean ± SEM, n = 5 mice per group; two-way *ANOVA*, Group × Time $F_{(4, 40)} = 0.148$, $p = 0.9628$, Group $F_{(1, 40)} = 0.06308$, $p = 0.803$, Time $F_{(4, 40)} = 74.32$, $p < 0.0001$, Bonferroni *post-hoc*). **d,** Deletion of Aldh1a1 inhibits GABA release from Aldh1a1 neurons. Blue laser lights were delivered (horizontal bar) after 2-min baseline. A plot shows the normalized levels of DA to the baseline (100%) from the individual (circles) Aldh1a1$^{+/+}$ (blue) and Aldh1a1$^{-/-}$ (green) mice and the averages per group (triangles, mean ± SEM, n = 5 mice per group; two-way *ANOVA*, Group × Time $F_{(4, 40)} = 24.76$, $p < 0.0001$, Group $F_{(1, 40)} = 23.27$, $p < 0.0001$, Time $F_{(4, 40)} = 33.54$, $p < 0.0001$, Bonferroni *post-hoc*).

Supplementary Fig. 3

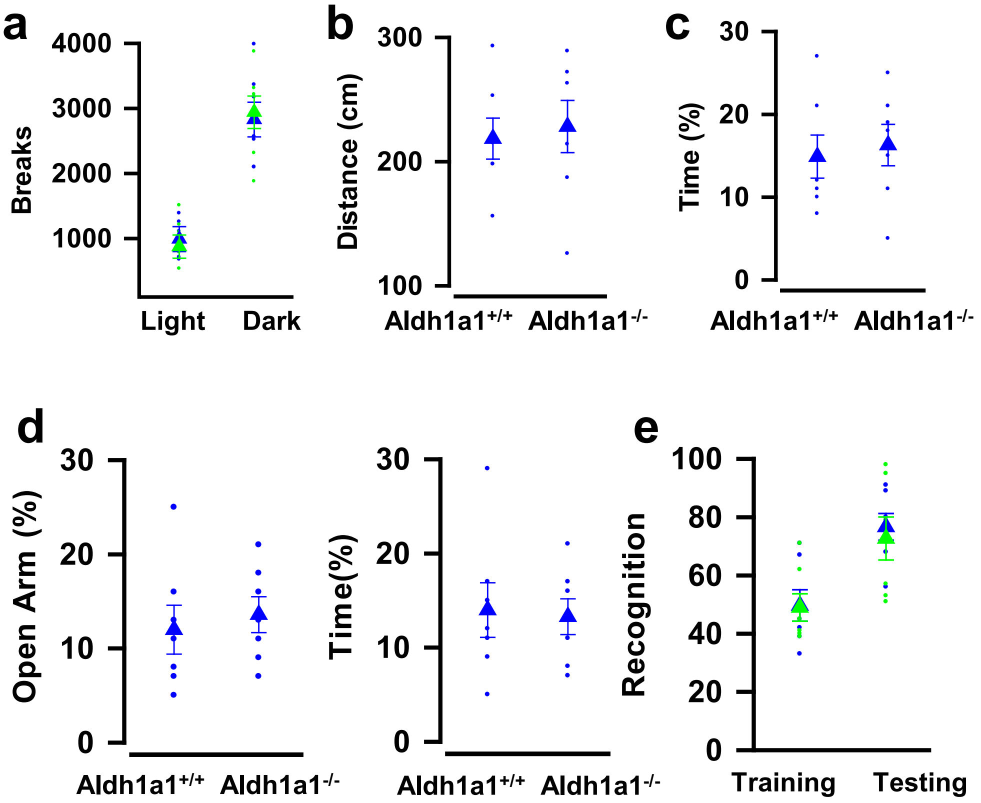

Supplementary Fig. 3. Aldh1a1$^{-/-}$ mice show normal motor activity. a-c, Aldh1a1$^{-/-}$ mice perform normally in light and dark cycles. Plots show the number of beam breaks in the individual mice (circles) and their averages per group (triangles, **a**), the distance traveled (**b**), and the percentage of time stayed in the central, **c**) of home cage .**d**, Aldh1a1$^{-/-}$ mice perform normally in elevated plus maze. Plots show open arm entries in the individuals (circles) and their averages (triangles) and the percentage of time staying in the central. **e,** Behavioral tests was performed with a 2-hr delay between the training and test sessions. Recognition index in the individual Aldh1a1$^{+/+}$ (blue) and Aldh1a1$^{-/-}$ (green) mice (circles) and their averages (triangles) was close to 50% during training sessions, showing that mice had no bias toward any of the objects and/or locations. During the test sessions, both groups of mice showed a significant preference for exploring the novel object.

Supplementary Fig. 4

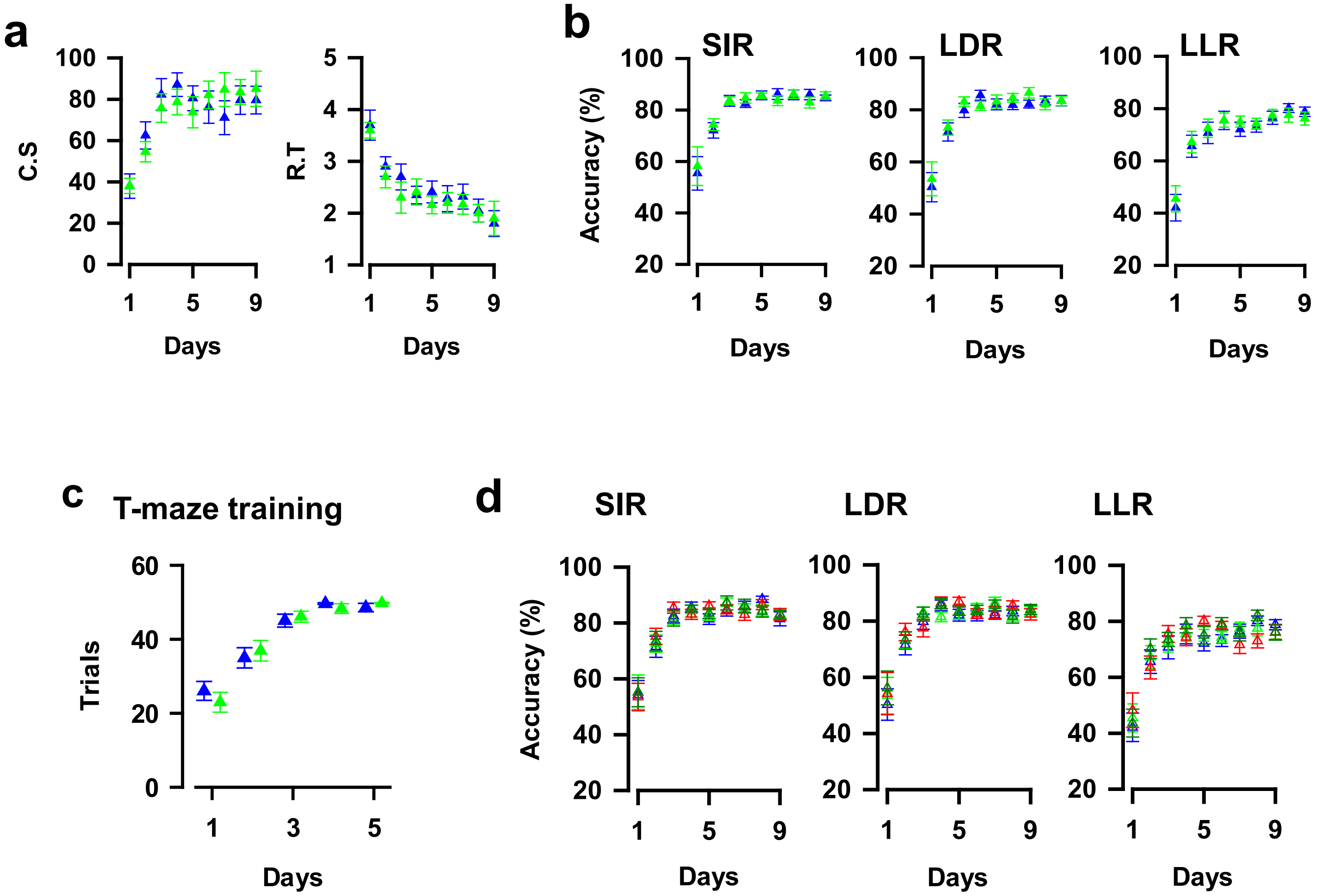

Supplementary Fig. 4. Deletion of Aldh1a1 produces no effects on motivation for reward. a, Plots showing the correct scores (C.S) and the reaction times (R.T) of Aldh1a1$^{+/+}$ (blue) and Aldh1a1$^{-/-}$ (green) mice at each day of the probe trials (mean ± SEM, n = 9 mice/group). **b,** Plots showing the accuracy of Aldh1a1$^{+/+}$ (blue) and Aldh1a1$^{-/-}$ (green) mice at each day of SIR, LDR, and LLR (mean ± SEM, n = 9 mice/group). **c,** Both Aldh1a1$^{+/+}$ (blue) and Aldh1a1$^{-/-}$ (green) mice performed similarly in completion of the training schedule (perform 50 trials per hours). **d,** Plots showing the accuracy of Aldh1a1$^{+/+}$ mice with the expression of eAldh1a1 (blue) or tdT (green) and Aldh1a1$^{-/-}$ mice with the expression of eAldh1a1 (red) or tdT (dark green) at each day of of SIR, LDR, and LLR (mean ± SEM, n = 9 mice/group).

Supplementary Fig. 5

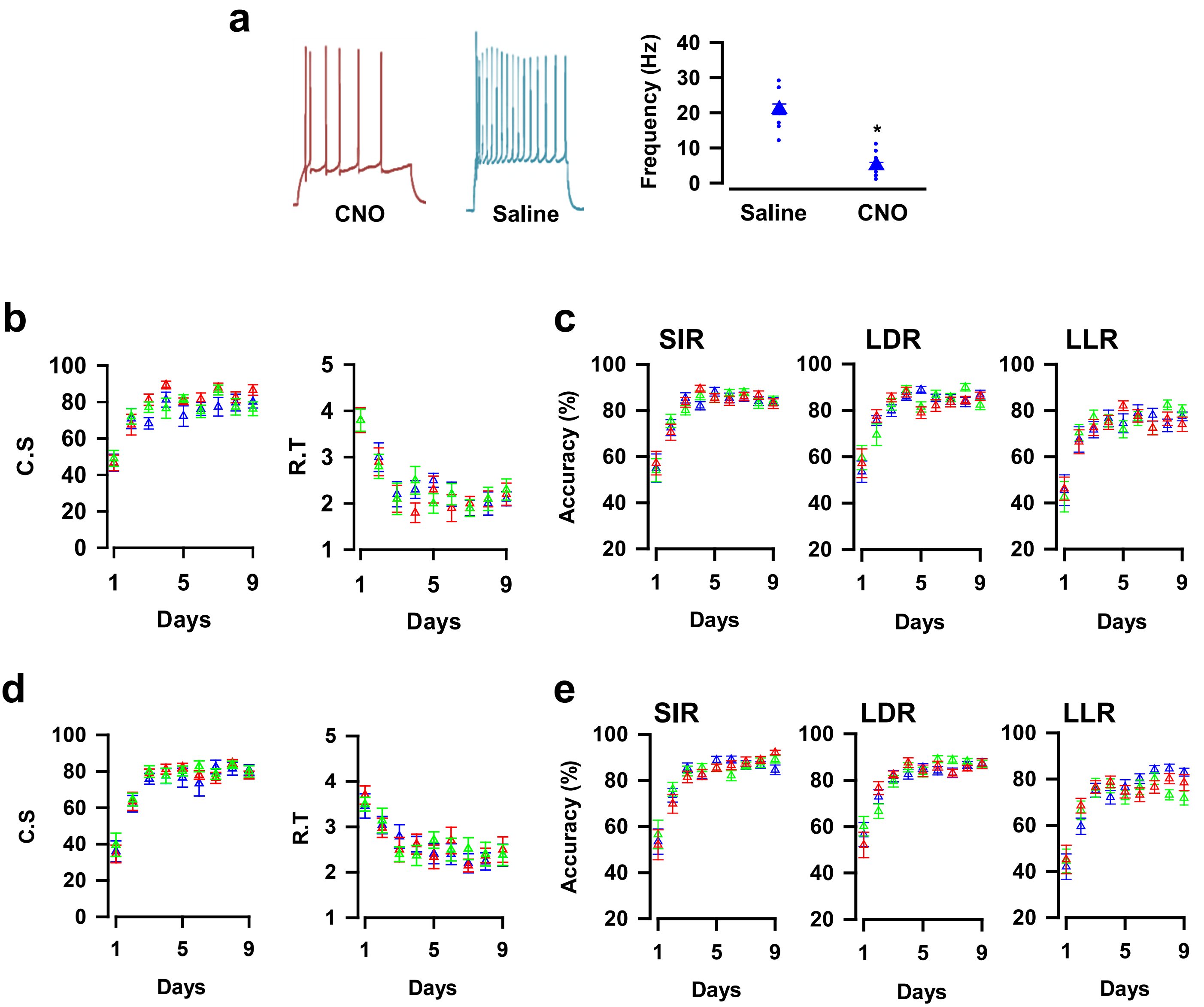

Supplementary Fig. 5 Chemogenetic silencing synaptic transmission$^{\mathbf{Aldh1a1 \rightarrow EGNIS}}$. a, Application of CNO reduces the frequency of action potential firings in response to depolarizing currents in Aldh1a1$^{Gi\text{-}ChR2}$ neurons. A plot shows the individual (circles) and the averages (triangles) per group (mean ± SEM, n = 11 recordings/ 6 mice/group, $p < 0.0001$, *t*-test) given with CNO or saline. **b**, Plots show C.S and R.T of Aldh1a1^{tdT} mice given CNO (blue) or Aldh1a1^{Gi} mice given with CNO (red) or saline (green) at each day of the probe trials (mean ± SEM, n = 9 mice/group). **c**, Plots show Accuracy of Aldh1a1^{tdT} mice given CNO (blue) or Aldh1a1^{Gi} mice given with CNO (red) or saline (green) at each day of SIR, LDR, and LLR (mean ± SEM, n = 9 mice/group). **d,** Plots show C.S and R.T of Aldh1a1$^{+/+}$-EGNIS^{Gi} mice given CNO (blue) or Aldh1a1$^{-/-}$ –EGNIS^{Gi} mice given with CNO (green) or saline (red) at each day of the probe trials (mean ± SEM, n = 9 mice/group). **e**, Plots show Accuracy of Aldh1a1$^{+/+}$-EGNIS^{Gi} mice given CNO (blue) or Aldh1a1$^{-/-}$ –EGNIS^{Gi} mice given with CNO (green) or saline (red) at each day of SIR, LDR, and LLR (mean ± SEM, n = 9 mice/group).

Supplementary Fig. 6

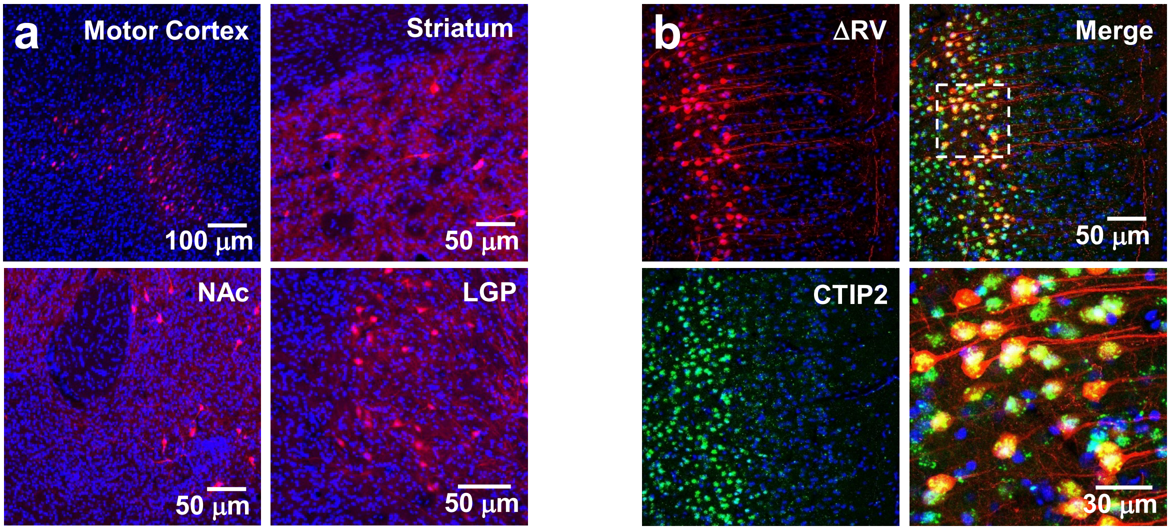

Supplementary Fig. 6. **Expression of RV in presynaptic neurons of Aldh1a1 neurons. a,** tdTomato was detected in several brain regions after the infection of ΔG-rabies virus (RV) in Aldh1a1 neurons, including motor cortex, striatum, nucleus accumbens (NAc), and lateral globus pallidus (LGP). **b,** Labeling of ΔRV-expressing neurons (red) in mPFC with anti-CTIP2 (a marker for cortical layer 5b neurons, green).

Supplementary Fig. 7

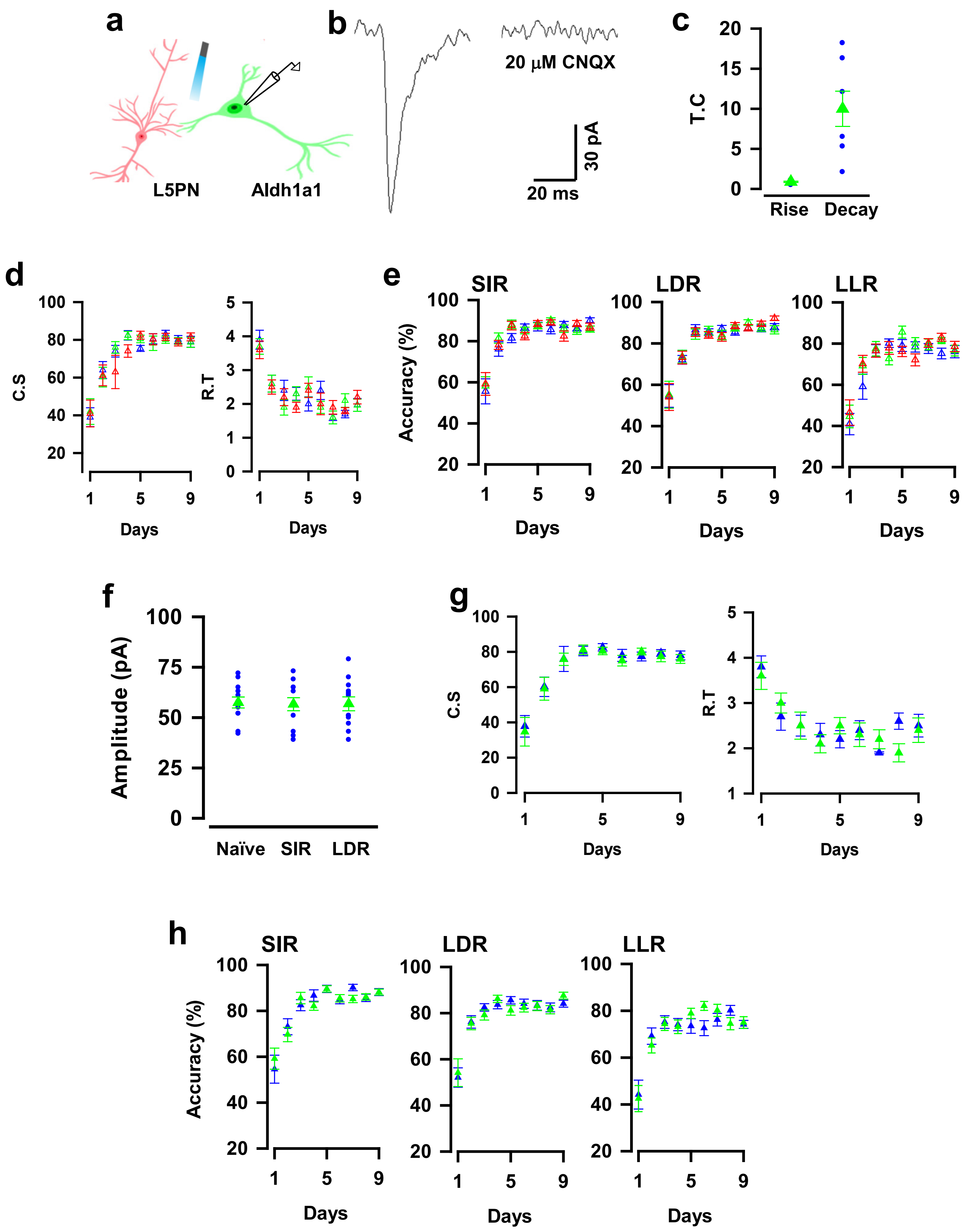

Supplementary Fig. 7. Glutamate excitatory synaptic transmission$^{L5PN \rightarrow Aldh1a1}$. a, Whole-cell patch clamp recordings in GFP-expressing Aldh1a1 neurons, and EPSCs were evoked by delivery of blue laser lights onto ChR2-expressing L5PN. **b,** Representative EPSCs at holding potential of -70mV were sensitive to CNQX. **c,** A plot shows rise and decay time constants (T.C) of EPSCs from the individual recordings (circles) and the averages (triangles, mean ± SEM, n = 7 recordings). **d**, Plots show C.S and R.T of L5PN^{tdT}-Aldh1a1^{ChR2} mice (blue) or L5PN^{Gi}-Aldh1a1^{GFP} mice (green) or L5PN^{Gi}-Aldh1a1^{ChR2} mice (red) at each day of the probe trials (mean ± SEM, n = 9 mice/group). **e**, Plots show Accuracy of L5PN^{tdT}-Aldh1a1^{ChR2} mice (blue) or L5PN^{Gi}-Aldh1a1^{GFP} mice (green) or L5PN^{Gi}-Aldh1a1^{ChR2} mice (red) at each day of SIR, LDR, and LLR (mean ± SEM, n = 9 mice/group). **f,** Plots show mean amplitudes of IPSC-GABA of naïve, SIR and LDR groups from the individual recordings (circles) and the averages (triangles, mean ± SEM, n = 12 recordings, $p > 0.5$, one-way *ANOVA*). **g**, Plots show C.S and R.T of mice given with AP5 (green) or saline (blue) at each day of the probe trials (mean ± SEM, n = 9 mice/group). **h**, Plots show Accuracy of mice given with AP5 (green) or saline (blue) at each day of SIR, LDR, and LLR (mean ± SEM, n = 9 mice/group).

Supplementary Fig. 8

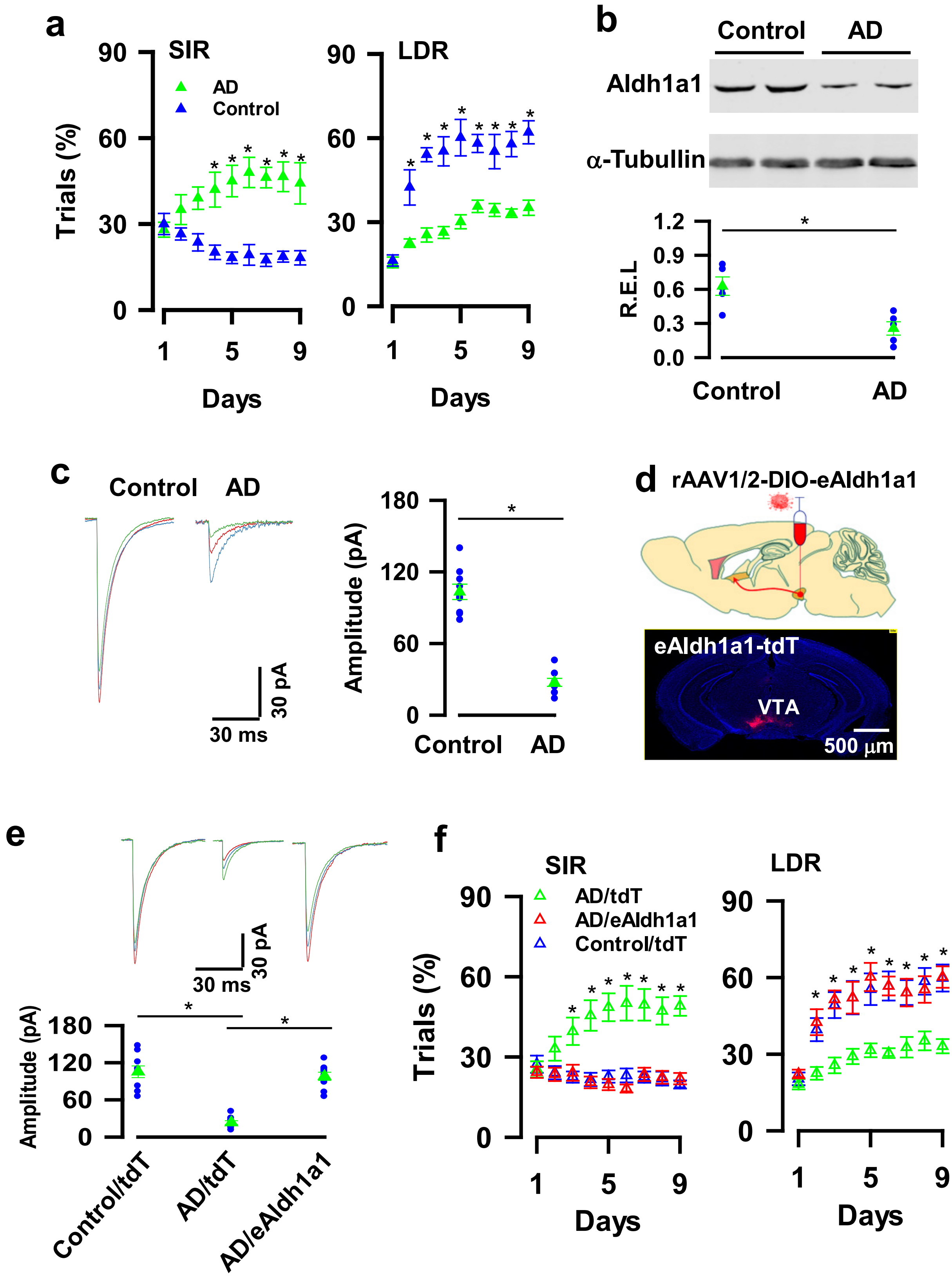

Supplementary Fig. 8. Dysfunction of Aldh1a1→EGNIS synaptic transmission causes impulsive behaviors in AD mice. a, AD mice show impulsive behaviors. Plots show the percentage of the correct trials with the behavioral options for SIR, or LDR in control (blue) and AD (green) mice (mean ± SEM, n = 9 mice/group) at each day of the probe. **b**, AD mice show a reduction of Aldh1a1 expression in Aldh1a1 neurons. Aldh1a1 neurons were purified from control and AD mice at 5 months old of age. Proteins from purified Aldh1a1 neurons were blotted and normalized to the respective α-Tubullin, defined as relative expression level (R.E.L, mean ± SEM, n = 5 mice/group). **c**, AD mice show dysfunction of Aldh1a1→EGNIS synaptic transmission. Three representative traces of IPSCs from EGNIS of control and AD mice are evoked are evoked by deliveries of blue laser lights onto axon fibers of $Aldh1a1^{ChR2}$ neurons. A plot shows the amplitudes of IPSCs from the individual control and AD mice (circles) and their averages per group (triangles, mean ± SEM, n = 9 mice/group). **d**, Expression of exogenous Aldh1a1 (eAldh1a1) in Aldh1a1 neurons of AD mice. AD mice were crossed with Aldh1a1-CRE mice, resulting in the production of AD/Aldh1a1-CRE mice. The rAAV1/2-DIO-eAldh1a1-tdT or the rAAV1/2-DIO-tdT virus particles were injected into VTA, resulting in the expression of Aldh1a1-tdT or tdT in Aldh1a1 neurons. **e**, Expression of eAldh1a1 restores Aldh1a1→EGNIS synaptic transmission in AD mice. Three representative traces of IPSCs from EGNIS of control and AD mice with the expression of tdT or eAldh1a1 are evoked by deliveries of blue laser lights onto axon fibers of $Aldh1a1^{ChR2}$ neurons. A plot shows the amplitudes of IPSCs from the individual (circles) and their averages per group (triangles, mean ± SEM, n = 9 mice/group). **f**, Expression of eAldh1a1 rescues delay of gratification in AD mice. Plots show the percentage of the correct trials with the behavioral options for SIR (left), LDR (right) in control (blue) and AD mice with the expression of tdT (green) or eAldh1a1 (red, mean ± SEM, n = 9 mice/group) at each day of the probe.

Supplementary Fig. 9

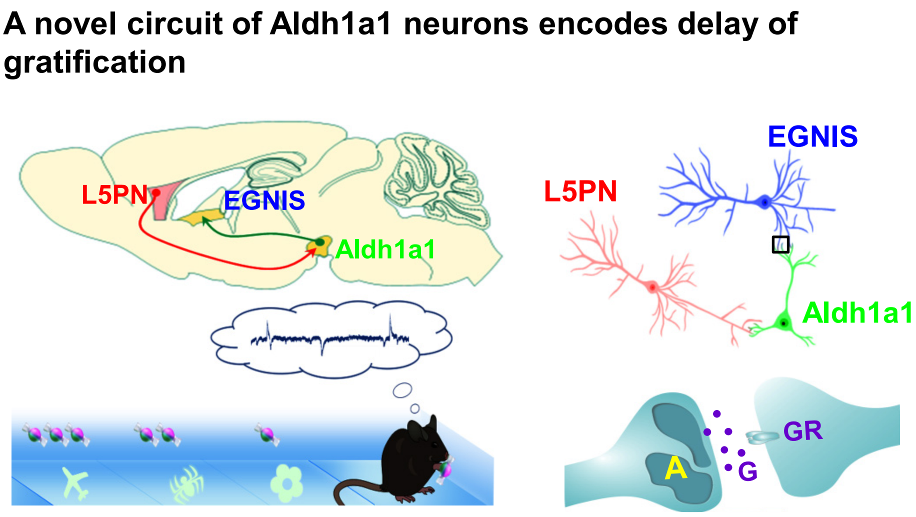

Supplementary Fig. 9. A novel circuitry of Aldh1a1 neurons encodes delay of gratification. Aldh1a1 neurons encode delay of gratification that measures self-control skills on value-directed decision making by projecting inhibitory synapses directly onto EGNIS and receiving excitatory synaptic inputs directly from L5PN. Synaptic transmission of Aldh1a1 neurons undergoes long lasting potentiation, a major form of synaptic plasticity, requiring activation of postsynaptic NMDA receptors. Application of NMDA receptor antagonist blocks this synaptic potentiation and impairs delay of gratification. Thus, delay of gratification depends on synaptic plasticity of Aldh1a1 neurons.

Supplementary Fig. 10

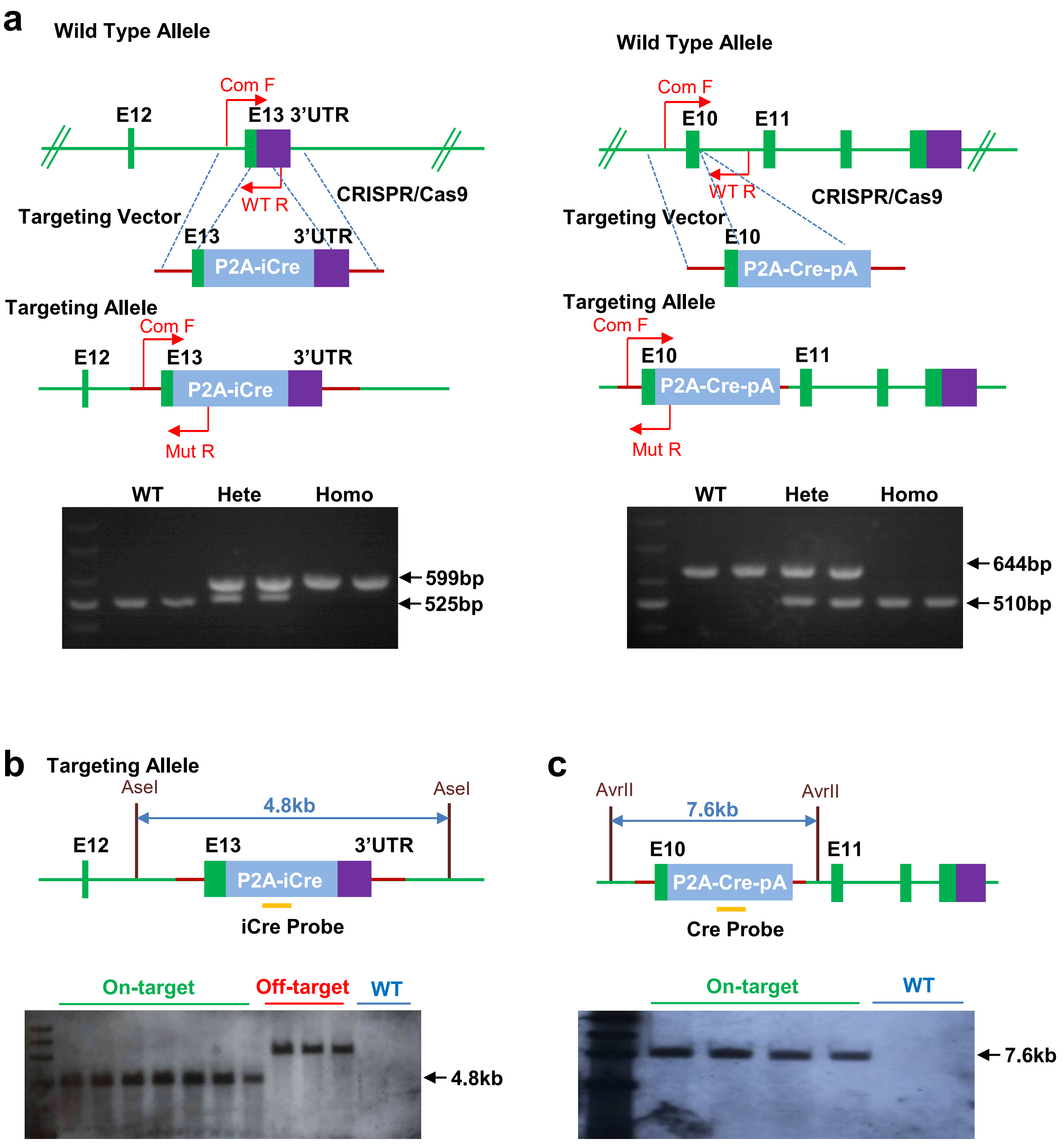

Supplementary Fig. 10. Vector design and genotype for generation of Aldh1a1-CRE mice. a, The strategy for generation of Aldh1a1-CRE (left) mice by inserting P2A-iCRE downstream of exon 13 regions and Aldh1a1$^{-/-}$-CRE (right) by inserting P2A-CRE-pA downstream of exon 10. Both Aldh1a1-iCRE and Aldh1a1$^{-/-}$-CRE mice were constructed with CRISPR/Cas9 strategy. The gRNA sequence is the following (5'-3'): ACGACTATGCTGGTTACTAT-AGG (Aldh1a1-CRE), GAGGAGGTAATGTTTCCACA-TGG (Aldh1a1$^{-/-}$-CRE). Cas9/sgRNA was cloned into the pCS vector (Biocytogen, China. www.bbctg.com.cn.). Guide-RNA, Cas9 mRNA, and donor DNA vector were microinjecting into zygotes from C57BL/6J mice. The genotype of the constructed mutant mice were validated by PCR and sequencing. The primers used for genotyping as the followings:

Aldh1a1-CRE-Common F: 5'-TGTCCACTGGCAAGTGTATGTTTGGC-3';

Aldh1a1-CRE-WT R: 5'-ATGACGGAGTTACAGAGAGCTTGCG-3';

Aldh1a1-CRE-Mut-R: 5'- GGCTTGCAGGTACAGGAGGTAG-3';

Aldh1a1$^{-/-}$-CRE-Common F: 5'- GTCGGAAGCCTCATCTTCGT-3';

Aldh1a1$^{-/-}$-CRE-WT R: 5'-CCCCTACAAAGCCAGTCCAG-3';

Aldh1a1$^{-/-}$-CRE-Mut R: 5'- GTTGTTCAGCTTGCACCAGG-3'.

b, Validation of on-target versus off-target effects in the generation of Aldh1a1-CRE mice. Southern blots from 10 F1 mutant and 2 control mice reveal that off-target effects were introduced in 3 F1 mutant mice, which were then excluded for the studies. **c**, The absence of off-target effects in the generation of Aldh1a1$^{-/-}$-CRE mice**.** Southern blots from 4 F1 mutant and 2 control mice reveal the absence of off-target effects introduced by CRIPR/Cas9 in the all F1 mutant mice.

Supplementary Fig. 11

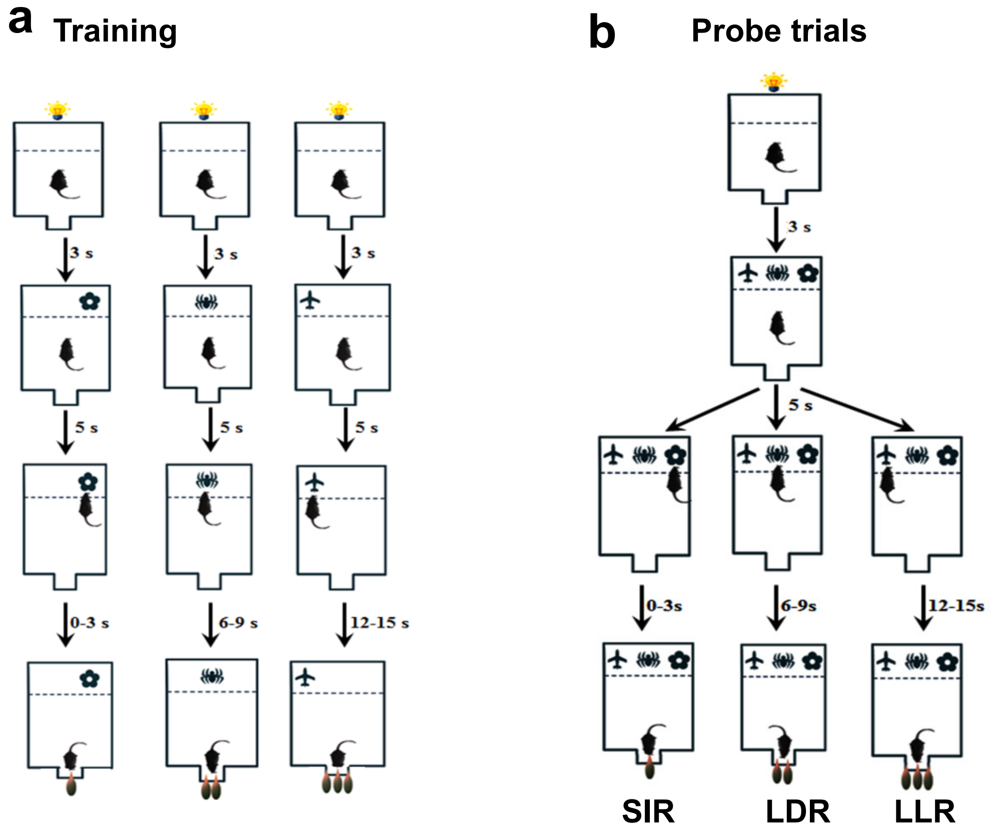

a, b, The behavioral tests comprised of training session (**a**) and probe trials (**b**). In the training sessions, each trial was started when a house light on. After 3 s, one of the three cue symbols was displayed on the touchscreen for 5 s. Mice were required to nose-poking this symbol within 5 s and collected a contingency reward with a specific delay. After successfully trained (>75% accuracy), mice were subjected to the probe trials, in which mice were required to freely choose among three symbols that were displayed on the touchscreen for 5 s.
